# Physical nanoscale conduit-mediated communication between tumour cells and the endothelium modulates endothelial phenotype

**DOI:** 10.1038/ncomms9671

**Published:** 2015-12-16

**Authors:** Yamicia Connor, Sarah Tekleab, Shyama Nandakumar, Cherelle Walls, Yonatan Tekleab, Amjad Husain, Or Gadish, Venkata Sabbisetti, Shelly Kaushik, Seema Sehrawat, Ashish Kulkarni, Harold Dvorak, Bruce Zetter, Elazer R. Edelman, Shiladitya Sengupta

**Affiliations:** 1 Harvard-MIT Division of Health Sciences and Technology, Cambridge, Massachusetts 02139, USA; 2 Massachusetts Institute of Technology, Cambridge, Massachusetts 02139, USA; 3 Department of Medicine, Brigham and Women’s Hospital, 65 Landsdowne Street, Room 317, Boston, Massachusetts 02115, USA; 4 Harvard Medical School, Boston, Massachusetts 02115, USA; 5 Department of Pathology, Beth Israel Deaconess Medical Center, Boston, Massachusetts 02215, USA; 6 India Innovation Research Center, New Delhi 110092, India; 7 Program in Vascular Biology and Department of Surgery, Children’s Hospital, Boston, Massachusetts 02115, USA; 8 Dana Farber Cancer Institute, Boston, Massachusetts 02115, USA

## Abstract

Metastasis is a major cause of mortality and remains a hurdle in the search for a cure for cancer. Not much is known about metastatic cancer cells and endothelial cross-talk, which occurs at multiple stages during metastasis. Here we report a dynamic regulation of the endothelium by cancer cells through the formation of nanoscale intercellular membrane bridges, which act as physical conduits for transfer of microRNAs. The communication between the tumour cell and the endothelium upregulates markers associated with pathological endothelium, which is reversed by pharmacological inhibition of these nanoscale conduits. These results lead us to define the notion of ‘metastatic hijack’: cancer cell-induced transformation of healthy endothelium into pathological endothelium via horizontal communication through the nanoscale conduits. Pharmacological perturbation of these nanoscale membrane bridges decreases metastatic foci *in vivo*. Targeting these nanoscale membrane bridges may potentially emerge as a new therapeutic opportunity in the management of metastatic cancer.

## Abstract (web summary)

Cancer cells and stromal cells have been shown to pass cellular information between each other via exosomes. Here, the authors demonstrate that cancer cells can communicate with endothelial cells through nanoscale membrane bridges, and demonstrate that microRNAs are passed through these nanobridges, which modulates endothelial cell phenotype.

## Graphical abstract

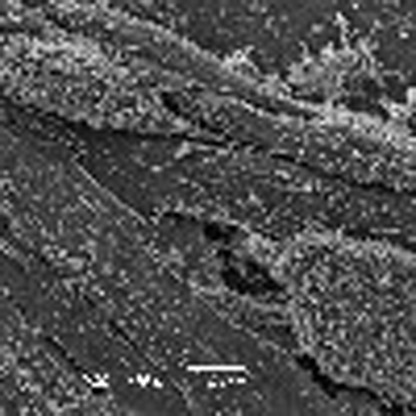

Angiogenesis and metastasis are the two key inflection points during tumour progression and are associated with a negative outcome^1^,^2^. Although angiogenesis is a critical, early driver of tumour growth^3^, metastasis is the final step in the progression of tumour accounting for >90% of cancer-related mortality^4^. Communication between cancer cells and the endothelium is the hallmark of both these processes^5^,^6^, and the cancer cells and the endothelium mount a dynamic regulation on each other via such communication^7^.

The cancer cell–endothelial communication in the context of angiogenesis has been extensively explored^8^. For example, paracrine signalling via growth factors is well established^9^,^10^. Similarly, in a recent study, communication between cancer and endothelial cells through tumour-secreted microRNAs (miRNAs) packaged in microvesicles was implicated in angiogenesis. Antagomirs that target this miRNA-mediated signalling were shown to inhibit angiogenesis and reduce tumour burden^11^. In contrast, the communication between tumour cells and endothelium in the context of metastasis is less explored.

Metastasis is the culmination of a cascade of events, including invasion and intravasation of tumour cells, survival in circulation, extravasation and metastatic colonization^4^. Multiple studies have reported a dynamic interaction between the metastatic tumour cell and the target organ, mediated by cytokines^4^,^12^ or by exosomes that can prime metastasis by creating a pre-metastatic niche^13^. Interestingly, the interactions between cancer cells and endothelium in the context of metastasis, which occurs during intravasation, circulation and extravasation, remains less studied. Cancer cell-secreted soluble factors can induce retraction of endothelial cells and the subsequent attachment and transmigration of tumour cells through the endothelial monolayers^14^,^15^. Recently, studies indicate a more intricate communication between cancer cells and the endothelium. For example, a miRNA regulon was found to mediate endothelial recruitment and metastasis by cancer cells^16^. Similarly, exosome-mediated transfer of cancer-secreted miR-105 was recently reported to disrupt the endothelial barrier and promote metastasis^17^. We rationalized that a better understanding of cancer–endothelial intercellular communication, primarily during extravasation, could lead to novel strategies for inhibiting metastasis^18^.

Recently, nanoscale membrane bridges, such as tunnelling nanotubes (TNTs) and filopodias, have emerged as a novel mechanism of intercellular communication^19^. For example, specialized signalling filopodia or cytonemes were recently shown to transport morphogens during development^20^. Similarly, TNTs, which unlike filopodia have no contact with the substratum^21^, were shown to facilitate HIV-1 transmission between T cells, enable the spread of calcium-mediated signal between cells and transfer p-glycoproteins conferring multi-drug resistance between cancer cells^22^,^23^,^24^,^25^. TNTs were also recently implicated in trafficking of mitochondria from endothelial to cancer cells and transfer miRNA between osteosarcoma cells and stromal murine osteoblast cells, and between smooth muscle cells and the endothelium^26^,^27^,^28^. However, whether similar intercellular nanostructure-mediated communication can be harnessed by cancer cells to modulate the endothelium is not known.

Here we report that metastatic cancer cells preferentially form nanoscale intercellular membrane bridges with endothelial cells. These nanoscale bridges act as physical conduits through which the cancer cells can horizontally transfer miRNA to the endothelium. We observe that the recipient endothelial cells present an miRNA profile that is distinct from non-recipient endothelial cells isolated from the same microenvironment. Furthermore, the co-cultures of cancer and endothelial cells upregulate markers associated with pathological endothelium, which is inhibited by pharmacological disruption of the nanoscale conduits. Additionally, the pharmacological inhibitors of these nanoscale conduits can decrease metastatic foci *in vivo*, which suggests that these nanoscale conduits may potentially emerge as new targets in the management of metastatic cancer.

## Results

### Cancer cells form nanoscale bridges with the endothelium

**Figure 1:**
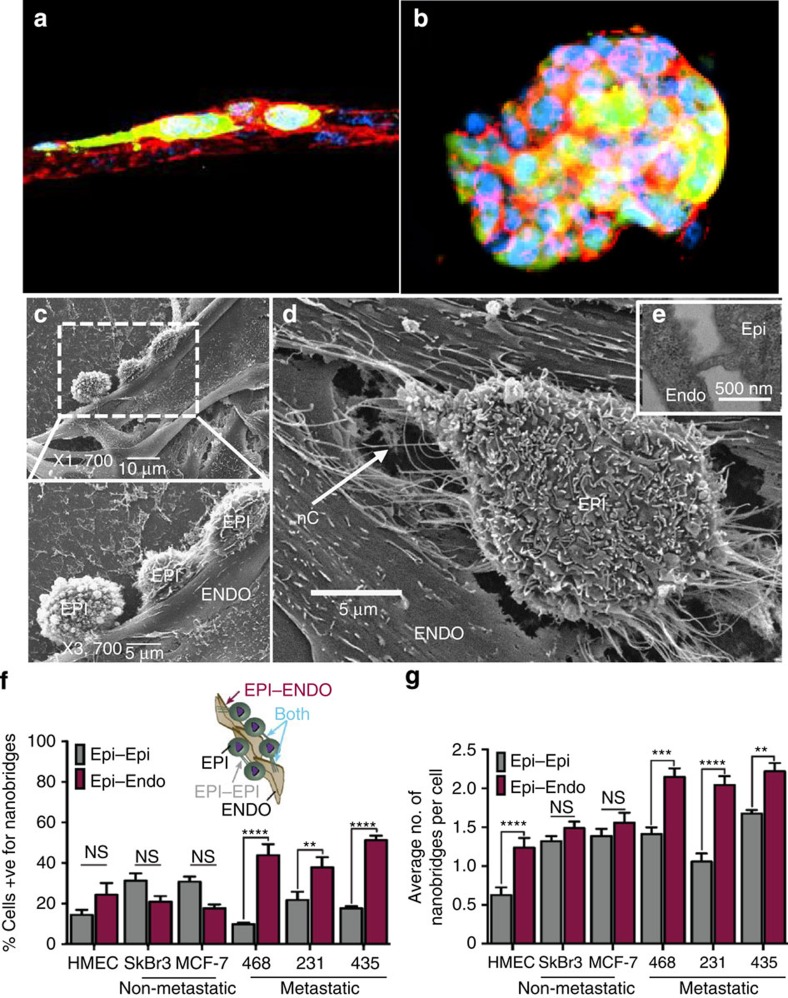
Nanoscale structures physically connect metastatic cells and the endothelium. (**a**) Representative image of MDA-MB-231 cancer cells exhibiting an invasive phenotype in the presence of preformed endothelial tubes in co-culture. (**b**) Representative image of a mammosphere typically formed by MDA-MB-231 cells when cultured on 3D tumour matrix in the absence of endothelial cells. MDA-MB-231 cells were loaded with CFSE. Actin was labelled with rhodamine phalloidin and nuclei were counterstained with DAPI. (**c**) A representative SEM of epithelial (EPI) MDA-MB-231 cells aligning on HUVEC (ENDO) tubules in the co-culture. Lower panel shows higher magnification. (**d**) SEM image reveals nanoscale membrane bridges connecting (nCs) metastatic breast cancer (EPI) cells and endothelial vessels (arrows). (**e**) A representative transmission electron micrograph shows intercellular connectivity through the nanoscale membrane bridge between MDA-MB-231 and an endothelial cell. (**f**) A cartoon represents the types of homotypic and heterotypic intercellular nanoscale connections that an epithelial cell may form in the presence of endothelial tubules. Highly metastatic (MDA-MB-468, MDA-MB-231 or MDA-MB-435) or low metastatic (MCF7 and SkBr3) cancer cells were co-cultured with the endothelial tubes. Normal HMECs were used as control. Graphs show percentage of total population of epithelial cells that exhibit either homotyptic (Epi–Epi) or heterotypic (Epi–Endo) nanoscale connections and (**g**) average number of nanoscale connections formed per cell. Quantification analysis was done on >300 cells of each cell type. Data shown are mean±s.e.m. (*n*=6 replicates per study, with 2–3 independent experiments). **P<0.01, ***P<0.001 (analysis of variance followed by Bonferroni’s *post-hoc* test).

In a previous study, we had observed that quiescent endothelial cells can mute the proliferative and invasive phenotype of cancer cells^7^, suggesting that cancer cells need to dynamically modulate an inhibitory physiological endothelium to facilitate metastasis. Here we set up a simple experiment, where we added the metastatic breast cancer cells to preformed endothelial tubes on a three-dimensional (3D) tumour matrix. The cancer cells preferentially attached to the vascular network, acquired an elongated phenotype and invaginated into the endothelial network (Fig. 1a). This was in contrast to the phenotype reported earlier, where monocultures of breast cancer cells were typically found to form characteristic mammospheres on 3D matrices (Fig. 1b)^29^. These results indicated that the interactions between metastatic cancer cells and endothelial cells can create a ‘niche’ that facilitates an invasive phenotype in the cancer cells. Indeed, the change in phenotype of the cancer cells was consistent with previous observations wherein tumour cells adopted a spindle-shaped morphology to migrate through endothelial layers^15^. Previous studies have shown that the endothelial tubules formed in these studies represent an ‘inside-out’ model of a blood vessel^30^, indicating that the cancer cells are exposed to the thrombogenic apical side of an endothelial cell, that is, the current assay likely modelled cancer cell–endothelium interactions in the context of extravasation.

Scanning electron micrographs (SEMs) confirmed that the cancer cells preferentially attach to the endothelial tubules and acquire an elongated morphology (Fig. 1c). Interestingly, we observed nanoscale membrane bridges connecting the cancer and endothelial cells (Fig. 1d). The bridges were found to hover over the substratum, consistent with the phenotype associated with TNTs^19^. These connections between epithelial and endothelial cells are referred as heterotypic connections. These nanoscale membrane bridges had the dimensions of 290±20 nm in the short axis and 30.69±2.43 μm in the long axis (mean±s.e.m., *n*>300 cells), similar to membrane nanotubes^31^, and were significantly distinct from cytoskeletal projections such as lamellipodia (short axis: 4.99±0.23 μm; long axis: 12.90±1.79 μm; mean±s.e.m., *n*>300) (Supplementary Fig. 1A). Transmission electron microscopy analysis revealed that the nanoscale membrane bridges enabled continuous intercellular connectivity between the two cells (Fig. 1e). We next tested the ability of cancer cells of different metastatic grades to form these membrane nanoscale connections with the endothelium (Supplementary Fig. 1B-F). Primary normal human mammary epithelial cells (HMECs) were used as controls. Nanoscale membrane bridges could form between two epithelial cells, which we term as homotypic connections (Fig. 1f and Supplementary Fig. 1G). In addition, an epithelial cell could simultaneously form both heterotypic and homotypic connections. Highly metastatic breast epithelial cells (MDA-MB-468 and MDA-MB-231) and metastatic melanoma (MDA-MB-435) cells formed significantly more intercellular nanoscale membrane bridges compared with both HMECs and low/non-metastatic cells (MCF7 and SkBr3) in co-cultures with endothelial cells, quantified as % of total cancer cells with membrane nanoscale bridges and also the number of nanotubes per cell. Tumorigenic but non/low-metastatic breast cancer cells (MCF-7 and SkBr3) contained higher number of nanoscale connections/cell than HMECs in co-culture, although the increase was a result of enhanced homotypic connections. Interestingly, as compared with the non/low-metastatic cell lines, the highly metastatic cells were found to preferentially form heterotypic nanoscale connections (Fig. 1f,g and Supplementary Fig.1G). A similar phenotype was conserved when the metastatic breast cancer cells were co-cultured with primary human dermal microvascular blood and lymph endothelial cells. Although the average number of nanoscale connections per cancer cell was greater in the presence of primary vascular endothelial cells, a greater percentage of the population of tumour cells exhibited these structures when cultured with lymphatic vascular cells (Supplementary Fig. 1H–L).

**Figure 2:**
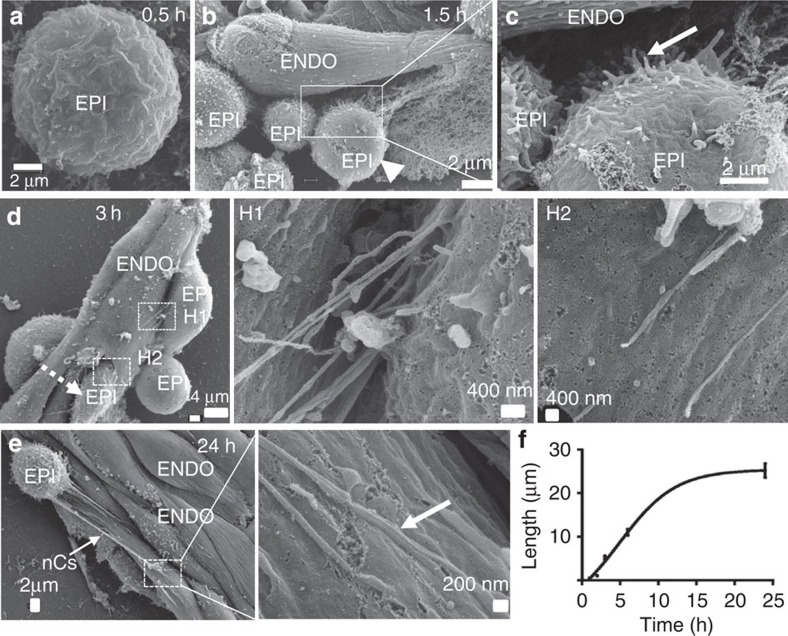
Temporal directed growth of heterotypic nanoscale membrane bridges from metastatic cancer cells to the endothelium. (**a**–**e**) Representative SEM images show temporal growth of the nanoscale bridges from the epithelial cells towards the endothelial cells over a 24-h period. (**c**) High-resolution image of the region highlighted by the box in **b** shows directionality of growth from the cell surface in close proximity to the endothelium (open arrow) but not the opposing pole (closed arrow). (H1 and H2) High-magnification images of highlighted regions in **d** show the growth of the nanoscale connections that can both hover over or attach to the substratum, finally fusing with endothelial cells as seen in **e** high magnification by 24 h. (**f**) Graph shows the quantification of the growth of the nanoscale structures over time. (*n*>300 cells, 6 replicates per study).

We next monitored the kinetics of formation of these intercellular nanostructures. The co-cultures of MDA-231 and endothelial cells were fixed at defined time points and imaged using an SEM. Consistent with previous observations, the cancer cells were found to attach with the endothelial cells, preferably in regions with additional cancer cells (Fig. 2). Interestingly, time-lapse analysis revealed that the nanoscale membrane projections develop from the metastatic cancer cells, from the surface in closest proximity to the endothelial tube, within 1.5 h of establishing the co-culture and evolving into stable intercellular structures over a 24-h period. Limited projections were observed from the opposite pole (away from endothelial cells) of the cancer cell (Fig. 2b), indicating that the formation of these structures occurred in a directed non-stochastic manner, consistent with a functional role. The peak lengths were reached between 15 and 20 h in co-culture (Fig. 2f).

### The nanoscale bridges are composed of cytoskeletal elements

**Figure 3:**
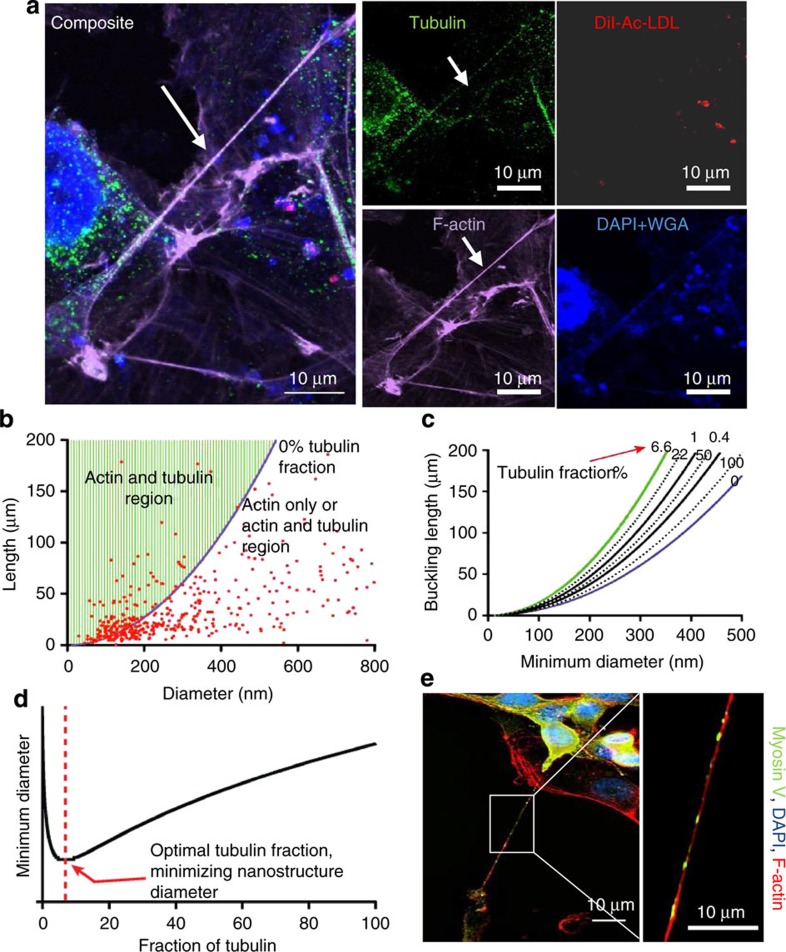
Structure and function of the heterotypic intercellular nanoscale membrane bridges. (**a**) Representative images show the heterotypic nanoscale membrane bridges are composed of both F-actin and α/β-tubulin cytoskeletal components. Co-cultures were stained with α/β-tubulin antibody (green) and phalloidin (purple) to label actin, and counterstained with DAPI (nuclear)+WGA (plasma membrane) (blue). Endothelial cells were labelled with DiL-Ac-LDL (red). (**b**) Mathematical modelling of the structure of the nanoscale connections. The physical properties of actin filaments necessitate microtubules for projections of certain length scales. The maximum projection length for a given minimum diameter at the buckling limit is plotted for actin-only nanoscale structures (purple line). This curve is overlaid with the experimental length and diameter measurements (red dots) from the observed thin projections measured in these studies. Projections containing only actin or projections containing both actin and tubulin can exist to the right of the curve (purple line). However, actin-only projections cannot exist to the left of the curve (green region). (**c**) The effect of incorporating tubulin in these projections. The maximum length is plotted against the minimum diameter for varying fractions of tubulin incorporated in the nanoscale projection. Addition of microtubules to the projections increases the overall flexural rigidity, shifting the curves left of the actin-only limit (purple line), thus allowing for longer and thinner nanoscale connections. However, owing to the larger radius of microtubules (4 × radius of actin filaments), there is an optimal fraction of tubulin (green line) that can be incorporated into the projection before the effect is reversed. (**d**) The optimal fraction of microtubules is about 6.6% (red dashed line) to maximize nanostructure flexural strength, while minimizing thickness. (**e**) Representative confocal image shows the presence of myosin V motor proteins within the intercellular nanostructure (inset shows higher magnification). Scale bar 10 μm.

To characterize the compositional structure of the nanoscale membrane bridges connections, we labelled the co-culture with phalloidin to delineate the actin filaments, 4,6-diamidino-2-phenylindole (DAPI) and wheat germ agglutinin (WGA) to stain the nucleus and cell membrane, respectively, and used immunolabelling for tubulin. As shown in Fig. 3, intercellular nanoscale connections were continuous membranous structures composed of actin supported by tubulin cytoskeletal components (Fig. 3a and Supplementary Fig. 2A). Analysis of a population of the nanoscale membrane bridges revealed that ∼70% were composed of both actin and tubulin, while the remaining were composed of only actin (Supplementary Fig. 2A). Previous studies have reported that membrane bridges or nanotubes could comprise only actin, as well as both actin and tubulin^19^. Indeed, extrapolating an established ‘filopodial’ mathematical model (Supplementary Discussion) to the observed structures indicates that at the observed mean diameter, a majority of the nanoscale membrane bridges, and especially the longer ones, should comprise both actin and tubulin, consistent with the fact that tubulin is critical for increasing flexural rigidity of the nanostructures at the observed length and diameter scales (Fig. 3b–d). However, owing to the larger radius of tubulins (4 × radius of actin filaments), there is an optimal fraction of tubulin (∼6.6%) to maximize nanostructure flexural strength while minimizing thickness. In addition, immunocytochemistry revealed a punctate Myosin V motor protein expression within these nanostructures (Fig. 3e), consistent with the previous observation of Myosin V in TNTs^31^. Myosin V motors are known to transport cargo progressively along actin filaments^32^. Taken together with the increased propensity of metastatic cells to form heterotypic nanoscale connections and the directionality of growth towards the endothelium, these compositional observations suggested that these membrane nanostructures could function as an intercellular highway enabling transfer of materials from the cancer cells to the endothelium.

### Nanoscale bridges act as conduits for communication

**Figure 4:**
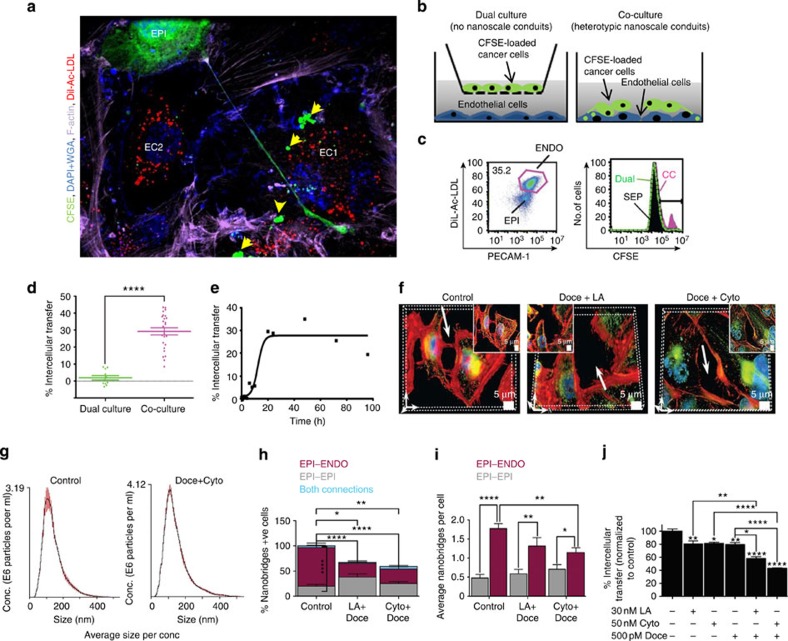
The nanoscale membrane bridges act as conduits for intercellular communication between cancer and endothelial cells. (**a**) Confocal image of nanoscale membrane bridge-mediated transfer of cytoplasmic contents. CFSE (green)-loaded MDA-MB-231 cells were co-cultured with the Dil-Ac-LDL (red)-labelled HUVECs. Transfer of the CFSE dye was observed after 24-h co-culture. CFSE dye can be seen within HUVEC cells (yellow arrow). Tumour cells can form a nanobridge with a distal endothelial cell (EC1) than an endothelial cell (EC2) in close proximity. (**b**,**c**) Cartoon shows the experimental design, where dual cultures control for vesicle-mediated intercellular transfer. FACS plot show gating for sorting endothelial cells from the co-cultures using dual staining for DiI-Ac-LDL and PECAM-1, and then quantification for CFSE transfer in the isolated endothelial cells. (**d**) Graph shows quantification of FACS analysis, highlighting increased transfer of CFSE to endothelial cells in the co-culture. (*N*>100,000 events, *n*=36 replicates, 3 replicates per study). (**e**) Graph shows the temporal kinetics of nanoscale connection-mediated intercellular transfer of CFSE from MDA-MB-231 cells to the endothelium (*n*=2 studies, 3 replicates per study). (**f**) Effect of small molecule inhibitors of cytoskeletal components on membrane nanobridges. (**g**) Graphs show treatment with vehicle (control) or a low-dose combination of docetaxel and cytochalasin do not affect the exosome shedding (*n*=2 independent studies). (**h**,**i**) Graphs show the effect of pharmacological inhibitors on the formation of heterotypic and homotypic nanoscale bridges (arrows). (*n*=2 studies, 6 replicates per study). (**j**) Graph shows the effect of pharmacological inhibitors on intercellular transfer of CFSE to endothelial cells from cancer cells (*n*=10 studies, 3 replicates per study). Data shown are mean±s.e.m. (*P<0.05, **P<0.01, ****P<0.001, analysis of variance followed by Bonferroni’s *post-hoc* test).

As the first step, to test the hypothesis that the nanoscale membrane bridges indeed facilitate intercellular communication from the cancer cells to endothelium, we loaded MDA-MB-231 breast cancer cells with a cell-impermeable dye, carboxyfluorescein succinimidyl ester (CFSE), before adding them to a co-culture with DiI-Ac-LDL-labelled endothelial cells. De-convolved volume 3D rendering of the co-cultures revealed transfer of cytoplasmic CFSE within the nanoscale membrane bridges connecting the cancer cells and endothelial cells (Fig. 4a and Supplementary Fig. 2B). Interestingly, although gap junctions have been reported to mediate intercellular transfers between cancer and endothelial cells^33^, here we observed intercellular transfer between distant cancer and endothelial cells that were not in direct physical contact except via the nanoscale membrane bridges (Fig. 4a). We next validated the intercellular transfer using flow cytometry. As a control, CFSE-loaded cancer cells and the endothelial cells were grown in the top and bottom chambers of a Boyden assay, respectively (dual culture), which allowed media contact and exosomes to cross through the 0.4-μm pores but did not allow direct physical contact, that is, no nanoscale membrane bridges could form between the cells (Fig. 4b and Supplementary Fig. 2C). In addition, we used a membrane with 3 μm pores, which allow both exosomes and larger vesicles to pass through (Supplementary Fig. 2C). The endothelial cells were first flow sorted from the dual/co-cultures using double labelling for DiI-Ac-LDL and platelet endothelial cell adhesion molecule-1 (PECAM-1) (Fig. 2c). The sorted endothelial cells were then analysed for CFSE and the subset of endothelial cells positive for CFSE was then quantified as a percentage of the total sorted endothelial cell population (Fig. 4c) as a measure of transfer from cancer cells. Indeed, we did observe intercellular transfer when the cells were separated in the Boyden assay (in both 0.4 and 3 μm pores), consistent with exosome- and extracellular vesicle-mediated transfer. Interestingly, as seen in Fig. 4d, <5% of the endothelial cells were found to be CSFE+ve in the dual culture (Boyden assay) as compared with ∼30% of the endothelial cells being labelled as CFSE+ve when isolated from the co-cultures (after subtracting background signal from both conditions). It is possible that this transfer seen in the co-culture study includes the basal transfer arising from exosome- or gap-junction-mediated transfer. Similar results were described in the TNT-mediated transfer of p-glycoproteins, where two cells separated by a membrane with 0.4 μm pores exhibited a basal transfer consistent with exosome-mediated transfer as opposed to higher levels of transmission when TNTs were present^26^. Furthermore, the temporal quantification of intercellular transfer of CFSE revealed that the peak is reached between 24 and 36 h, lagging behind the kinetics of formation of these nanoscale structures (Fig. 4e). The lag in transfer kinetics is consistent with the notion that the nanoscale connections are not fully functional at the early stages of formation. A similar transfer was observed between the metastatic breast cancer cells and primary human vascular and lymphatic endothelial cells (Supplementary Fig. 3). In addition to CFSE, the nanostructures facilitated the transfer of nanoparticles (quantum dots) and proteins (green fluorescent protein) (Supplementary Fig. 4A–E). Interestingly, we did not observe a similar communication between metastatic tumour cells and vascular smooth muscle cells, further emphasizing the specificity of the communication between cancer cells and the endothelium (Supplementary Fig. 5). These results indicated that the nanoscale bridges act as conduits for communication from cancer cells to endothelial cells.

### Effect of pharmacological inhibition of nanoscale bridges

We next performed loss-of-function studies to further validate the above hypothesis. As the nanoscale membrane bridges were composed of building blocks that could not be genetically knocked down without causing lethality, we harnessed a pharmacological approach, using docetaxel and latrunculin A or cytochalasin D, to perturb the two major components of the intercellular nanoscale membrane bridges, that is, tubulin and actin, respectively. A key limitation of these inhibitors is that they can exert nonspecific anti-mitotic effects leading to cell death. We therefore first performed titration studies to establish the threshold concentration below which the inhibitors did not exert any nonspecific effect on cell migration, proliferation or apoptosis (Supplementary Fig. 6A–F). As shown in Fig. 4f, at concentrations below the threshold, pretreatment of metastatic cells with a combination of docetaxel (500 pM) with latrunculin A (30 nM) or cytochalasin D (50 nM) disrupted the formation of the heterotypic intercellular nanostructures. Drug treatment inhibited the total number as well as the length of intercellular nanostructures, suggesting that the inhibitors prevent initiation and growth of the nanostructures. It should be noted that at these concentrations the inhibitors did not disrupt the basal transfer between HMECs or non-metastatic MCF7s and endothelial cells but reversed the increased intercellular transfer observed between the metastatic MDA-MB-231 cells and the endothelium to the basal level (Supplementary Fig. 7), which suggested that the basal transfer could occur via a mechanism independent of the formation of the nanoscale connections. Indeed, at these concentrations, drug treatment did not inhibit the shedding of exosomes from the cancer cells (Fig. 4g and Supplementary Fig. 7B), suggesting that the basal transfer could possibly be mediated via exosomes. This was further validated in a similar study, where cytochalasin disrupted nanotubes in phaechromocytoma cells but had no effect on endocytosis or phagocytosis^34^. Interestingly, the inhibitors reduced the heterotypic epithelial–endothelial intercellular nanostructures to a greater degree compared with homotypic epithelial–epithelial connecting nanostructures (Fig. 4h,i). Recent reports have indicated that some homotypic nanoscale connections could rise as vestiges of cytokinesis during cellular division^35^. In contrast, heterotypic connections can only develop *de novo*. To test this further, we stained the epithelial cells with Cep55, which labels the cytokinetic bridges. As shown in Supplementary Fig. 6G, the epithelial cells were found to be connected via nanoscale membrane as well as cytokinesis bridges (positive for both Cep55 and actin). Treating the cells with low-dose docetaxel (500 pM)+cytochalasin D (50 nM) disrupted the nanoscale membrane bridges but the cytokinesis bridges were found to be intact. This is consistent with our *in vitro* viability studies, where the cells were viable at these concentrations. Indeed, at a higher concentration (docetaxel 50 nM+cytochalasin D 50 nM), both the cytokinesis bridges as well as the nanoscale membrane bridges were inhibited. These results indicate that the pharmacological inhibitors, at the appropriate titrated concentration where it perturbs the *de novo* origins of the heterotypic membrane bridges without impacting cytokinesis, could be powerful tools to exquisitely dissect the functions of the heterotypic nanoscale connections between the metastatic cancer cell and the endothelium without the confounding nonspecific effects of a global knockdown of cytoskeletal components. The pharmacological disruption of nanoscale membrane bridges between metastatic cancer cells and the endothelium inhibited the transfer of CFSE from the former to the latter, validating that the nanostructures can indeed act as conduits for intercellular communication (Fig. 4j).

### Nanobridges transfer miRNA from cancer cells to endothelium

**Figure 5:**
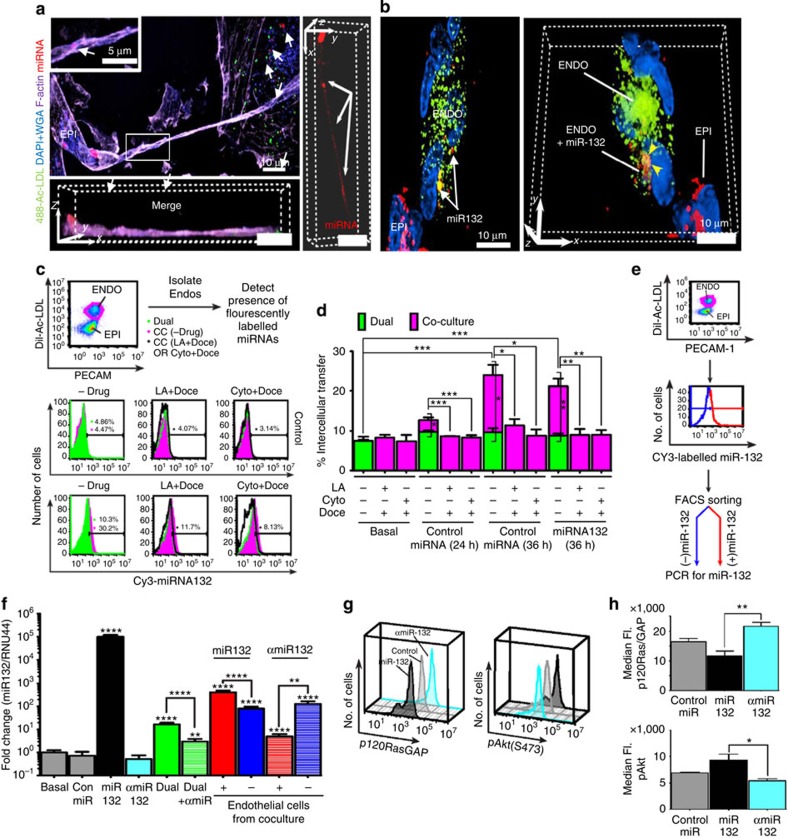
The nanoscale membrane bridges act as conduits for intercellular transfer of miRNA between cancer and endothelial cells. Representative confocal images show the transfer of Cy3-labelled miRNA from MDA-MB-231 cells (EPI) to endothelial cells (ENDO) at (**a**) 24 h and (**b**) 36 h of co-culture. Alexa Fluor 488-Ac-LDL (green)-labelled endothelial cells were co-cultured with Cy3-labelled miRNA-transfected MDA-MB-231. Co-cultures were counterstained with phalloidin (purple) and DAPI+WGA (blue). A 3D visualization shows the localization of miRNA within the nanoscale connections (white arrows), which act as conduits for horizontal transfer of miRNAs to endothelial cells. (**c**) Schema shows quantification of Cy3-labelled control miRNA and Cy3-labelled miR132 transfer between cancer cell and endothelium using flow cytometry. Endothelial cell populations were isolated from the co-cultures and percentage of miRNA+ve cells was determined. Dual cultures in Boyden chambers were included as controls. (**d**) Graph shows the effect of pharmacological disruption of nanoscale conduits on miRNA transfer. (**e**) Schema shows experimental design for reverse transcriptase–PCR-based detection of transferred miR-132 in endothelial cells under different experimental conditions. MDA-MB-231 cells transfected with miR-132 and α-miR-132 were co-cultured with endothelial tubes. FACS-isolated endothelial cell populations were analysed for the expression of miR-132. (**f**) Graph shows miR-132+ve cell populations (solid red) show 5 × increase compared with miR-132−ve populations (solid blue) (*P*<0.0001), whereas anti-miR-132+ve cells (striped red) show 26 × decrease in miR-132 expression (*P*<0.0001) compared with α-miR-132−ve cells (striped blue). Direct transfection of miR-132 (black) and α-miR-132 (light blue) in endothelial cells is used as positive and negative controls, respectively. Upregulation of miR-132 from baseline was observed in dual culture (solid green), which could be inhibited with anti-miR-132 (striped green). MiR-132 levels are increased compared with dual only in those cells that are positive for intercellular transfer. Fold change was determined compared with endothelial cell transfection with control miRNA (grey). (**g**) FACS analysis shows nanoscale bridges-mediated transfer of miRNAs leads to changes in p120RasGAP and pAkt (S473) expression downstream of the miR-132 pathway in endothelial cell populations isolated from co-cultures. (**h**) Graphs show p120RasGAP expression is decreased in the miR-132+ve cell populations and increased in the α-miR-132+ve cell populations, while further downstream miR-132 positively regulates pAkt expression. Data shown are mean±s.e.m. (*N*=2–5 independent studies, with 3 replicates per study, *P<0.05, **P<0.01, ***P<0.001, ****P<0.0001, analysis of variance followed by Bonferroni’s *post-hoc* test).

Although our study revealed the nanoscale membrane bridges could act as conduits for intercellular transfer, we rationalized that communication via the transfer of miRNAs from the cancer cells to the endothelium could result in the maximal amplification of signalling. Indeed, multiple studies have highlighted the role of miRNAs as signalling regulators in tumour cell migration and invasion^36^. For example, miR-132 was reported to be highly expressed in the endothelium of human tumours but was undetectable in normal endothelium. Furthermore, conditioned media from MDA-MB-231 cells was shown to upregulate miR-132 in endothelial cells^37^. As a proof-of-concept, we assessed whether the nanoscale membrane bridges can act as a physical conduit for transfer of miR-132 from metastatic cancer cells into endothelial cells. Cy3-labelled control miRNA or miR-132 was transfected in the metastatic MDA-MB-231 cells, which were then used to establish the co-cultures with endothelial cells. As shown in Fig. 5a,b (Supplementary Fig. 8), Cy3-labelled miRNAs were detected within the nanoscale bridges and were transferred to the endothelial cells. To further validate the transfer, we quantified Cy3-labelled miRNA in endothelial cells by flow cytometry (Fig. 5c,d). As a control, the cancer and endothelial cells were separated in dual chambers of a Boyden assay, which revealed a baseline transfer of Cy3-labelled miRNAs from the cancer cells to the endothelium that remained constant between 24 and 36 h. Indeed, a previous study reported that the kinetics of exosome-mediated miRNA transfer between MDA-MB-231 and endothelial cells starts by 4 h and peaks by 24 h^17^. In contrast, a significant increase in Cy3-labelled miRNAs in the endothelial cells was observed over baseline by 36 h of co-culture (Fig. 5d). Interestingly, pretreating the cancer cells with a combination of docetaxel and cytochalasin or latrunculin A, at concentrations previously established to inhibit the formation of the nanoscale membrane connections without affecting exosome shedding, reduced the elevated miRNA levels in the endothelial cells in the co-cultures but had no effect on basal transfer (Fig. 5d). This further validated that basal transfer is probably mediated by exosomes, whereas the nanoscale membrane bridges play a critical role as conduits for enhancing miRNA-mediated communication between the metastatic cancer cells and the endothelium.

The above results were further confirmed using PCR reaction to quantify the expression of translocated miR-132 in the endothelial cells. Endothelial cells were isolated from the co-culture and were further flow sorted into Cy3-miR-132 +ve and −ve populations (Fig. 5e). As compared with basal expression of miR-132 in naive endothelial cells, the dual culture in Boyden chamber, which allowed media contact and exosome transfer, resulted in elevated levels of miR-132 in the endothelial cells. A similar increase in miR-132 was also observed in endothelial cells that were negative for Cy3 label in the co-culture experiment and thus served as an internal control (Fig. 5f). This increase in miR-132 in the control arms is consistent with earlier reports that cancer cell-secreted factors can induce the expression of miR-132 (ref. 37). In comparison, the Cy3+ve endothelial cell population from the co-culture exhibited significantly higher levels of miR-132. In addition, the treatment of the cancer cells with miR-132 antagomirs inhibited the expression of miR-132 in endothelial cells. Furthermore, as shown in Fig. 5g, endothelial cells receiving miR-132 showed a decrease in expression of downstream p120RasGAP and an increase in pAkt (S473), consistent with earlier observations^37^, which was reversed with anti-miR-132 or pretreatment of cancer cells with low-concentration cytochalasin and docetaxel (Fig. 5h and Supplementary Fig. 9). These results indicate that the nanoscale conduits might emerge as a significant mechanism of intercellular miRNA transfer besides exosomes.

### Nanobridge-mediated transfer alters endogenous miRNA profile

**Figure 6:**
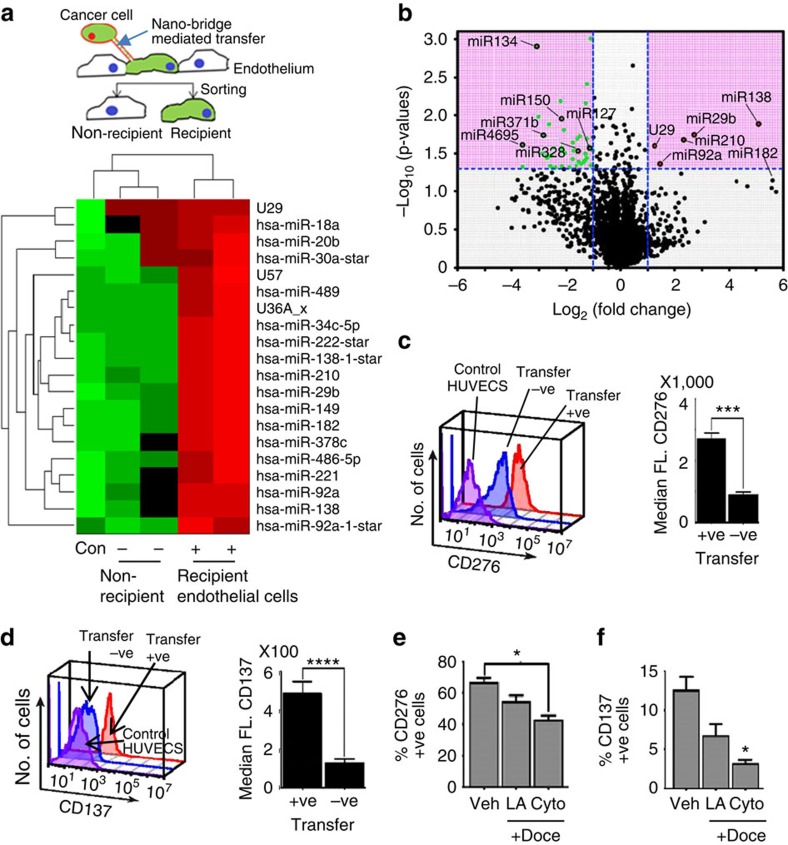
Cancer cell–endothelial intercellular transfer alters the endogenous miRNA profile and phenotype of recipient endothelial cells. (**a**) Schema shows the experimental design. CFSE(green)-loaded MDA-MB-231 cells were co-cultured with the Dil-Ac-LDL (red)-labelled HUVECs. A miRNA microarray was used to evaluate the transport of endogenous miRNAs. The intercellular CFSE-transfer−ve and -transfer+ve endothelial cells were sorted from the same pool. The heat map shows potential miRNA candidates that were significantly upregulated in the cells receiving transfer of intercellular contents from the cancer cells. HUVECs that were not exposed to cancer cells were used as a baseline control. (**b**) The volcano plot shows the statistically significant upregulated (red) and downregulated (green) miRNAs in the HUVEC cells that received intercellular transfer compared with those that did not receive transfer. (**c**,**d**) Sorting of the endothelial cells from the co-cultures with MDA-MB-231 cells reveal higher expression of tumour endothelial markers CD137 and CD276 in intercellular transfer +ve endothelial cell populations compared with intercellular transfer −ve cells. (**e**,**f**) Pharmacological inhibition of nanoscale tether formation reduced the expression of CD137 and CD276 in endothelial cells isolated from the co-cultures. Data shown are mean±s.e.m. (*n*=5 studies, with 3 replicates per study, ****P<0.0001, ***P<0.001, *P<0.05. Analysis of variance followed by Bonferroni’s *post-hoc* test).

Once we had established that the nanoscale membrane bridges do act as physical conduits for horizontal transfer of miRNA, we next tested whether such transfers can alter the endogenous miRNA profiles in the recipient endothelial cells. We set up a simple experiment, where CFSE-loaded MDA-MB-231 cancer cells were co-cultured with endothelial cells for 36 h and the latter were then sorted into CFSE+ve (recipient) and CFSE−ve (non-recipient) populations (Fig. 6a). The non-recipient (CFSE−ve) endothelial cells therefore served as an internal control, as both pools (recipient and non-recipient) were exposed to the same exogenous cancer cell-secreted factors. An additional control group was run, where endothelial cells were cultured without any exposure to tumour cells, and were considered as naive cells. miRNA profiling data did indicate that culturing tumour cells with endothelial cells can alter the miRNA signature of the latter. Both recipient and non-recipient endothelial cells from the co-cultures exhibited a distinct miRNA profile compared with naive endothelial cells (Fig. 6a), consistent with previous reports implicating cell-secreted growth factors, microvesicles and exosomes in modulating the miRNA regulome^11^,^37^. For example, miR-18a expression, which can be induced by vascular endothelial growth factor, was upregulated in endothelial cells isolated from the co-culture and predicts a poor prognosis in patients with breast cancer^38^,^39^. The interesting finding was the distinct miRNA profiles of the two pools of recipient and non-recipient endothelial cells. For example, the recipient endothelial cells exhibited a significant number of upregulated miRNAs (Fig. 6a,b), many of which have been implicated in activation of endothelium and/or metastasis^40^,^41^,^42^. For example, miR-92, belonging to the miR-17-92 cluster (Oncomir-1), has been implicated in cancer metastasis to lymph nodes^43^. Similarly, transfer of miR-210 from metastatic cancer cells to endothelial cells results in angiogenesis and metastasis^41^. Furthermore, upregulation of miRNA-182 and miR29b was observed in invasive and metastatic breast cancer^44^,^45^. We also observed several downregulated miRNAs in the recipient endothelial cells (Fig. 6b). For example, miR150, which can target vascular endothelial growth factor-A, and miR885, which has been implicated as a tumour suppressor, were downregulated in the recipient endothelial cells^46^,^47^. At a phenotypic level, the analysis of cell surface markers revealed an upregulation in the expression of CD276 (Fig. 6c) and CD137 (Fig. 6d) in the recipient endothelial cells as compared with non-recipient endothelial cells from the same co-culture. Pharmacological inhibition of the nanoscale conduit formation decreased the expression of CD137 and CD276 in endothelial cells in the co-culture (Fig. 6e,f).

### Nanoscale bridge-mediated intercellular transfer *in vivo*

**Figure 7:**
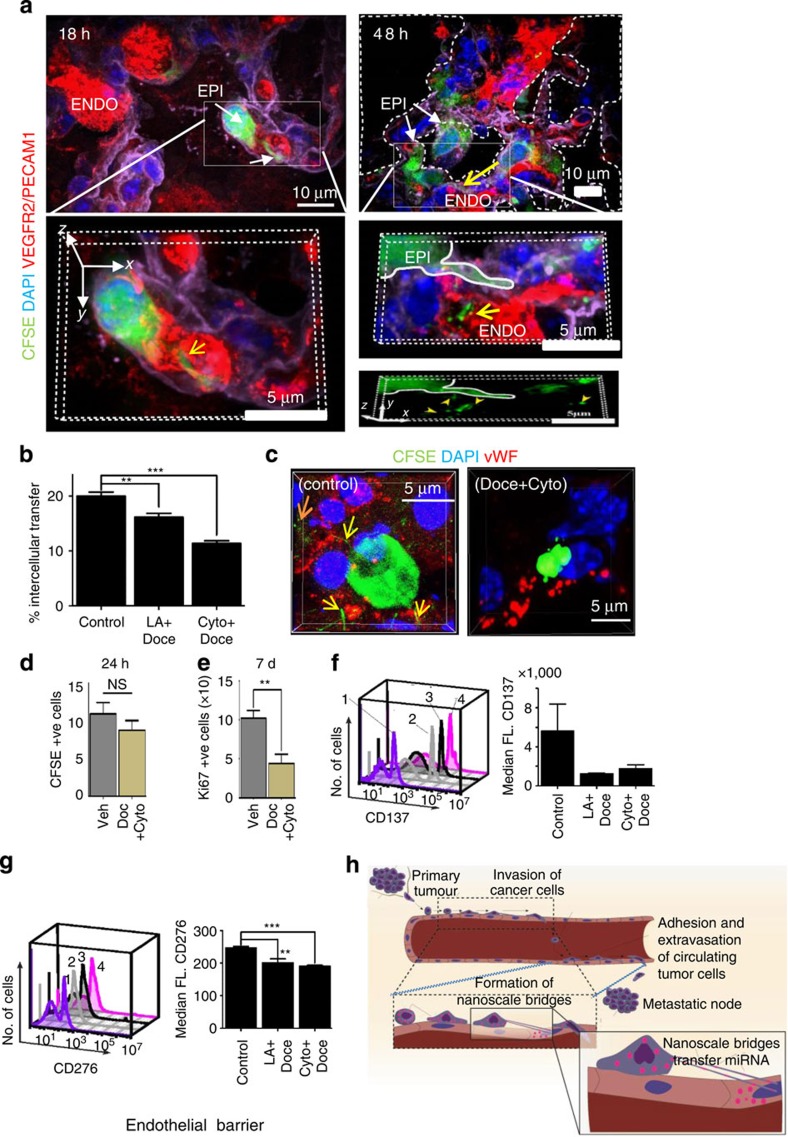
Nanoscale membrane bridge-mediated intercellular communication *in vivo*. (**a**) Three-dimensional confocal reconstructions demonstrate the nanoscale membrane bridges from CFSE-loaded MDA-MB-231 cancer cells transferring CFSE to the vascular endothelial growth factor receptor 2 (VEGFR2)/PECAM1-labelled endothelial cells (white solid outline). Examples of intercellular transfer are indicated with yellow arrows. Animals were injected with CFSE-loaded MDA-MB-231 cancer cells via the tail vein and the lungs were excised at defined time points to monitor cancer cell–endothelial interactions. (**b**) Graph shows the effect of pre-treatment of MDA-MB-231 cancer cells with low-dose pharmacological inhibitors of nanoscale membrane bridges on the transfer of CFSE to lung endothelial cells *in vivo*. Endothelial cells were isolated from the mouse lungs 48 h post injection. Heterotypic intercellular transfer was quantified using FACS. (**c**) Confocal images of lung sections from balb/c mice treated with CFSE-loaded 4T1 metastatic breast cancer cells show transfer of CFSE to vWF-labelled lung endothelial cells in vehicle treated but not in the case of drug pretreatment that inhibits the formation of nanoscale connections. (**d**) Graph shows the number of CFSE+ve tumour cells in the lungs 24 h post injection, showing no significant (NS) difference between pretreated and vehicle-treated groups. (**e**) Graph shows a reduction in metastatic foci at day 7 in the pretreated, that is, inhibition of nanoscale connections, versus vehicle-treated groups. Data shown are mean±s.e.m. The total number of tumour cells over ten sections was quantified for each lung. Lungs were isolated from *n*=4 mice in each treatment group (**P<0.01 versus vehicle treated). (**f**,**g**) Isolation of lung endothelial cells and quantification of CD137 and CD276 expression using FACS reveal decreased expressions in conditions where the injected MDA-MB-231 cells were pretreated with pharmacological inhibitors to disrupt the nanoscale connections. (1) Naive endothelial cells; (2) endothelial cells from cytochalasin+docetaxel-treated group; (3) docetaxel+Latrunculin A-treated group; and (4) endothelial cells from vehicle-treated MDA-MB-231 group. Data shown are mean±s.e.m. (*n*⩾3, **P<0.01, ***P<0.001 versus control, analysis of variance followed by Bonferroni’s *post-hoc* test). (**h**) Schematic illustration of potential role of nanoscale conduit-mediated intercellular heterotypic communication in metastatic progression. The horizontal transfer of cellular material, including miRNA, can alter the endothelial regulon and switch the endothelial barrier to a dysfunctional ‘enabling’ pre-metastatic niche.

We next studied whether the nanoscale membrane bridge-mediated intercellular communication between cancer cells and the endothelium occurs *in vivo*. We used well-established *in vivo* models that capture the extravasation step of metastasis^7^,^48^. MDA-MB-231 cells, which metastasize to the lungs, were injected intravenously (i.v.) in mice. The animals were killed at the specified time points and the interaction between the CFSE-loaded cancer cells and the endothelium of the lung vasculature was studied by confocal microscopy after immunolabelling the endothelial cells. As seen in Fig. 7a and Supplementary Fig. 10A, CFSE+ve cancer cells were visualized adjacent to the lung endothelium as early as 18 h post injection and an increasing number of cancer cells in close proximity were evident by 48 h, consistent with the *in vitro* observation that the cancer cells tended to cluster around an endothelial niche. Interestingly, intercellular transfer of CFSE to the endothelial cells was detected by 18 h (Fig. 7a). By 72 h, micrometastases were found in the lung parenchyma. To validate that the transfer of CFSE from cancer cells to the endothelial cells is indeed mediated by the nanoscale conduits, we pre-treated the CFSE-loaded cancer cells with low-dose pharmacological inhibitors to block nanobridge formation. Treatment-naive and pharmacological inhibitor-treated cancer cells were then injected i.v. into mice, which were killed at 48 h post injection. The lung endothelial cells were isolated using magnetic separation and were sorted into CFSE+ve and −ve population. As shown in Fig. 7b, pharmacological inhibition of nanoscale conduit formation resulted in a decrease in the intercellular transfer of CFSE compared with treatment-naive cancer cells.

Separately, we studied whether the pharmacological disruption of nanoscale conduit-mediated intercellular transfer perturbs the metastatic process. CFSE-loaded 4T1 breast cancer cells were injected i.v. in balb/c mouse. Consistent with previous reports^49^, 4T1 cells were found to metastasize to the lungs. As shown in Fig. 7c, nanoscale conduit-mediated CFSE transfer from cancer cells to lung endothelial cells was evident in the control group, which was abolished when the cancer cells were pretreated with the pharmacological inhibitors. Quantifying the number of tumour cells in lung cross-sections at an early time point (24 h post injection) revealed no significant differences between the two groups, suggesting that the ability of the cancer cells to remain in circulation is not altered by the treatment (Fig. 7d). In contrast, a significant reduction in the metastatic index, quantified as a function of Ki-67 staining (it is noteworthy that low-dose treatment did not inhibit proliferation index), was noted in the pharmacologically pretreated group on day 7, consistent with a reduced metastatic burden (Fig. 7e).

As our *in vitro* studies indicated phenotypic changes in the recipient endothelial cells compared with non-recipient cells, we performed an *in vivo* study, where CFSE-loaded MDA-MB-231 cells were injected into mice and the lung endothelial cells were isolated as described above at 48 h post injection using magnetic separation. The endothelial cells were then sorted into a CFSE+ve (that is, recipient) and CFSE−ve (that is, non-recipient) pools, and analysed for CD137 and CD276 expressions. The transfer-recipient endothelial cells exhibited higher expression of cell surface CD137 and 276 as compared with the non-recipient endothelial cells. In addition, pharmacological inhibition of nanoscale conduit formation reduced the expression of CD137 and CD276 on the lung endothelial cells (Fig. 7f,g and Supplementary Fig. 10B–D).

## Discussion

The complexity of regulatory tumour parenchyma–endothelial communication is increasingly being unravelled^7^,^50^. The altered phenotypic behaviour of the metastatic cancer cells in the presence of endothelial cells observed in this study, instead of forming classical mammospheres, is consistent with the emerging paradigm of modulatory tumour parenchyma–stroma communication and the creation of a pre-metastatic niche. Indeed, a recent study proposed the concept of the formation of a pre-metastatic niche mediated via metastatic cell-secreted exosomes, leading to vascular leakiness at the pre-metastatic sites^13^. Here we demonstrate that cancer cells form nanoscale membrane bridges, which can act as conduits for horizontal transfer of miRNA from the cancer cells to the endothelium, switching the latter to a pathological phenotype. Our findings reveal that the ability to form the nanoscale conduits with endothelial cells correlates with the metastatic potential of the cancer cell, and that the pharmacological perturbation of these nanoscale connections can lead to a reduction in the metastatic burden in experimental metastasis models. Together, our studies shed new insights into the tumour parenchyma–endothelial communication, adding depth to the emerging paradigm of the ability of a cancer cell to ‘hijack’ a physiological stromal cell for self-gain^13^.

Indeed, exosomes have emerged as an extensively studied mechanism of horizontal intercellular transfer of information^51^. However, a key distinction exists between the exosome-mediated versus the nanoscale membrane bridge-mediated intercellular communication. Although the former is stochastic, that is, it is unlikely the cancer cell has control over which cell will be targeted by a secreted exosome, the communication via nanoscale membrane bridges is deterministic, that is, the cancer cell can connect to a specific endothelial cell, which could be further away than the most proximal endothelial cell.

Although the aim of this study was to study the nanoscale membrane bridges as a mode of horizontal transfer of miRNAs from the metastatic cancer cells to the endothelium, and not to characterize a specific miRNA that are implicated in metastasis, many of the miRNAs, which were differentially regulated in the recipient endothelial cells, have previously been shown to regulate metastasis (Supplementary Discussion). For example, a downregulated miRNA, miR885, and an upregulated miR29 have both been implicated in directly suppressing the expression of CD276 (B7-H3)^52^, indicating that the overall increase in CD276 expression in recipient endothelial cells is probably an outcome of the altered miRNA regulome. Indeed, phenotypic overexpression of CD276 and CD137 is reported in tumour endothelium and correlates with metastasis^53^,^54^,^55^,^56^,^58^. Activation of CD137 can increase endothelial cell surface expression of adhesion molecules such as intercellular adhesion molecule-1, vascular cell adhesion molecule-1 and E-selectin, which can contribute to recruitment and extravasation of cells^53^,^54^,^55^,^56^,^58^. Although multiple mechanisms could play a role in the transition of an endothelial cell from a physiological to a pathological phenotype, the observation that pharmacological disruption of the nanoscale membrane bridges inhibits the increase in CD276 and CD137 expression both *in vitro* and *in vivo* suggests that the nanoscale membrane bridge-mediated cancer cell–endothelial communication contribute to the process. Indeed, not every endothelial cell is transformed, but as outlined in a recent study the alteration of a fraction of endothelial niche cells is sufficient to open ‘gates’ in these natural barriers for extravasation of cancer cells, thereby facilitating metastasis^17^.

In summary, our study add to the increasing repertoire of mechanisms that facilitate horizontal miRNA transfer in the metastatic setting. Our observations also open up many fundamental questions, which need to be addressed in future studies. For example, is there a heterogeneity of endothelial cells, that is, are there subsets of endothelial cells that attract the deterministic growth of the nanoscale membrane projections from cancer cells, and what are the drivers of the directed growth of these nanoscale bridges? Furthermore, there is significant heterogeneity in the subtypes of nanoscale membrane bridges, and the characterization and extent of involvements of these subtypes in metastasis needs to be elucidated. Similarly, we do see heterogeneity within a cancer cell population in their ability to form these nanostructures; what governs this heterogeneity? Interestingly, emerging insights that cancer cells with lower proliferation rate^59^ and enriched with lipid rafts form a higher number of intercellular nanoscale connections. These properties are typically associated with cancer stem-like cells^60^. This raises an interesting question whether the cancer cells that form nanoscale membrane bridges are more stem like? Indeed, further understanding of the nanoscale conduit-mediated intercellular communication can offer potential novel strategies for the management of metastatic disease, which is currently associated with dismal 5-year survival rates.

## Methods

### Cell culture

Human umbilical vein endothelial (HUVEC) cells (ATCC) were cultured on 0.1% gelatin in EBM-2 (Lonza) supplemented with bullet kit (Lonza) and 0.1% antibiotic/antimycotic (A/A) (Life Technologies). Human primary blood and lymph endothelial cells, collected from the plasma, were cultured on collagen (1:60) in MCDB 131 supplemented with 5% MVGS (Life Technologies), 1% L-alanyl-L-glutamine (Life Technologies) and 1% A/A. MDA-MB-231 (ATCC), MDA-MB-435 (ATCC) and MCF-7 (ATCC) were cultured in DMEM medium supplemented with 10% fetal bovine serum (FBS) and 1% A/A. MDA-MB-468 cells were cultured in DMEM medium supplemented with 5% FBS and 1% A/A. SKBR3 (ATCC) were cultured in McCoy’s 5A (Life Technologies) supplemented with 15% FBS and 1% A/A. HMEC cells (Life Technologies) were cultured in MEBM (Lonza) supplemented with MEGM bullet kit (Lonza) and 1% A/A. All cells were mycoplasma free. For pharmacological inhibition, cells were incubated with cytoskeletal pharmacological inhibitors Latrunculin A (Sigma), Cytochalasin D (Sigma) and Docetaxal (Sigma) in complete media for 24 h post 6–18 h of serum deprivation. Cell viability was quantified using MTS (3-(4,5-dimethylthiazol-2-yl)-5-(3-carboxymethoxyphenyl)-2-(4-sulfophenyl)-2H-tetrazolium) assay, where MDA-MB-231 cells were plated in a 96-well plate and drug treated for 24–48 h. MTS reagent (Life Technologies) was added to the sample and the plate was analysed using a BioTek Epoch Microplate Spectrophotometer. The results were validated using Alexa Fluor 488 Annexin V (Life Technologies) assay, where drug-treated MDA-MB-231 cells were incubated with endothelial cells, and imaged after 24 h. The fluorescence of each image was measured and compared between the different drug treatment groups.

### Co-culture

Endothelial cells were incubated with DiL-Ac-LDL reagent (1:100) (Life Technologies) in complete media for 1 h, plated in their respective media on 1:1 dilution Matrigel in PBS and incubated for 4–6 h (HUVEC and primary human dermal microvascular blood endothelial cells) or 24 h (primary human dermal microvascular lymph endothelial cells). Epithelial cells were loaded with CellTrace CFSE (Life Technologies), Qtracker® (Life Technologies), LysoTracker® (Life Technologies) or miRNAs (Life Technologies) according to the manufacturer’s specifications. The cells were added to the preformed vessels in their respective media in a 1:1 epithelial cell:endothelial cell ratio, incubated for defined time periods before further analysis.

### miRNA labelling and transfection

The Cy-3-labelled control miRNA was purchased from Life Technologies. miRNA-132 (Life Technologies) and αmiRNA-132 (Life Technologies) were labelled using Label IT miRNA Labeling Kit (Mirus) according to manufacturer’s protocol. Cells were transfected with Control miRNA (Life Technologies), miRNA-132 (Life Technologies) and αmiRNA-132 (Life Technologies). The miRNAs were transfected with siPORT NeoFX transfection reagent (Life Technologies) at a concentration of 50 nM and 1 × Opti-MEM I (Life Technologies). All transfections were completed according to the manufacturer’s protocols for 24 h.

### Fluorescence-activated cell sorting

Cells were fixed in 4% paraformaldehyde. Samples were stained in 100 μl of staining buffer (0.1% Sodium Azide (Sigma), 5% FBS, 1% BSA (Sigma) in PBS) with the following antibodies: CD31 (abcam, 1:100), monoclonal Anti-human LYVE-1-APC (R&D Systems,1:100), CD137 (Abcam, 1:50), CD276 (Abcam, 1:100), p120RasGAP (Santa Cruz Biotechnology, 1:100), or pAKT (Cell Signaling, 1:100). Quantification was done using Cflow Plus (or Flowjo) software.

### *In vivo* studies

MDA-MB-231 or 4T1 cells were injected i.v. into female CD1 nude or balb/c mice (4–6 weeks) respectively. Animals were sacrificed at defined time points and lungs were harvested. Lung metastasis was quantified by macroscopic evaluation of MDA-MB-231 lung nodules in Buijon’s solution on Day 3. Endothelial cells from lungs were isolated using magnetic CD31 beads followed by staining with CD137 or CD276 and sorted using a BD FACS Aria IIu SORP. All studies were conducted as per protocol approved by Harvard IUCAC.

### Immunohistochemistry

Samples were fixed with 4% paraformaldehyde (PFA) at room temperature for 15 min and washed with sodium borohydride (dissolved in PBS). Cells were stained with the following: rhodamine phalloidin (Life Technologies), Alexa-fluor 647 phalloidin (Life Technologies), α/β Tubulin antibody (Cell Signaling, 1:100), Myosin (Life Technologies, 1:100), WGA-CF405S conjugate (Biotium) and Alexa Fluor 647 Conjugate WGA (Life Technologies). Nuclei were counterstained with DAPI (Life Technologies). Paraffin-embedded sections were deparaffinized and antigen retrieval was carried out using sodium citrate buffer (10 mM sodium citrate, 0.05% Tween 20, pH 6.0) at 100 °C for 30 min. Samples were stained with rabbit anti-human CD31 (1:50), Von Willebrand Factor (1:300), Alexa Fluor 647 Conjugate WGA and DAPI (Life Technologies). For studying the effect of pharmacological inhibitors on cytokinesis bridges, MDA-MB-231 cells were treated with a combination of docetaxel (500 pM–50 nM) with cytochalasin D (50 nM) in complete media for 24 h post 6–18 h of serum deprivation. The cells were immunostained with primary rabbit anti-CEP55 antibody (1:250). For visualizing actin, the cells were immunostained with rhodamine phalloidin (Life Technologies-Invitrogen, USA). The imaging was performed using confocal microscopy.

### Scanning electron microscopy

Samples were fixed with 0.1 M sodium cacodylate (Sigma), 2% gluteraldehyde (Electron Microscopy Sciences), 3% PFA (Electron Microscopy Sciences), 5% sucrose buffer (Sigma) and 1% osmium tetroxide (pH 7.4) (Electron Microscopy Sciences). The samples were then dried in increasing concentrations of high-grade ethanol, followed by critical point drying using Autosamdri 815 critical point dryer and sputter coated using Cressington 208HR sputter coating with Au or Pt/Pd. Imaging was done on a Jeol 5600LV SEM, Zeiss EVO SEM or Zeiss FESEM Ultra55 microscope. For each image the total number of cancer cells, cancer cells with nanotubes, cancer cells without nanotubes, total number of nanotubes, total number of EPI–EPI membrane nanobridges, EPI–ENDO nanobridges, number of cells forming EPI–EPI nanobridges, EPI–ENDO nanobridges and number of cells positive for both EPI–EPI and EPI–ENDO nanobridges were counted. Length and width of the nanobridges were measured using the CarlZeiss TIF annotation editor. Width was measured at three different positions across the length of the nanobridges and the average width was calculated for the comparison of length and width of the nanobridges.

### Transmission electron microscopy

Cells were fixed in 2.5% gluteraldehyde, 3% PFA with 5% sucrose in 0.1 M sodium cacodylate buffer (pH 7.4), pelletted and post fixed in 1% OsO_4_ in veronal-acetate buffer. The cell pellet was stained in block overnight with 0.5% uranyl acetate in veronal-acetate buffer (pH 6.0), then dehydrated and embedded in Embed-812 resin. Sections were cut on a Reichert Ultracut E microtome with a Diatome diamond knife at a thickness of 50 nm, stained with uranyl acetate and lead citrate. The sections were examined using an FEI Tecnai spirit at 80 KV and photographed with an AMT CCD (charge-coupled device) camera.

### Imaging

Fluorescence imaging was performed on a Nikon Eclipse Ti camera (Nikon Instruments) with NIS Elements Imaging Software (3.10). Confocal fluorescence imaging was done on a PerkinElmer Ultraview Spinning Disk Confocal Microscope with Velocity acquisition software and Hammamatsu ORCA-ER CCD camera. Contrast and brightness parameter adjustments were applied across the whole image or equally across all the comparison groups when necessary. Quantification was done using NIS Elements Software; we measured the length of the complete and broken nanoscale connections, as well as other projections. *Z*-stack images were processed using the deconvolution software, to generate 3D reconstruction images.

### Mathematical model

A standard filopodial model was applied. Buckling force was calculated using the equation (1).




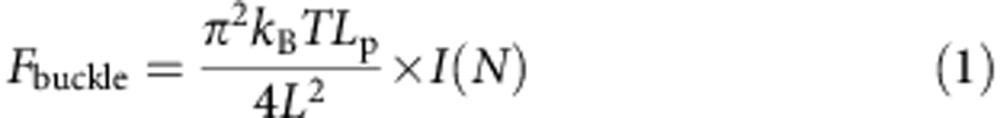




Where

*k*_B_: Boltzmann constant

*T*: temperature (body temperature)

*L*_p_: persistence length (microfilament/microtubule)

*L*: projection length

*N*: Total number of filaments

*I*: non-dimensional factor (
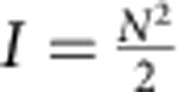
 in this mocel)

Buckling length was calculated using the equation described by equation (2).




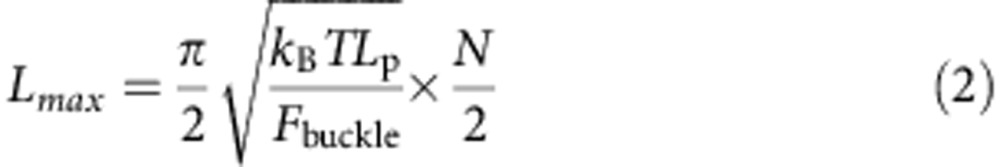




Projection persistence length was calculated using equation (3).




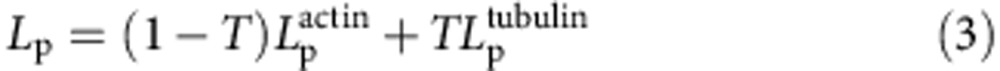




Where




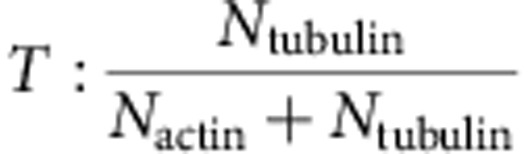




Minimum diameter of projections was calculated using equation (4)




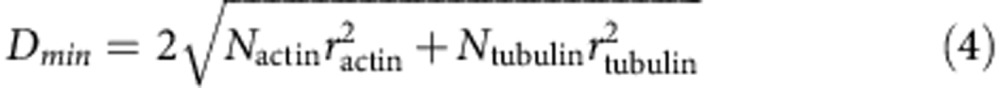




### Exosome analysis

MDA-MB-231 breast cancer cells were cultured in DMEM medium (supplemented with 10% exosome-depleted FBS (Exo-FBS) and 1% of antibiotic–antimycotic 100 × solution). To avoid the contamination from higher levels of exosomes in normal FBS, EXO-FBS (System Biosciences, Inc.) was used to culture the cells. The growth and morphologies of the cells cultured in Exo-FBS media was similar to the cells grown in normal FBS media, showing that the EXO-FBS do not have any effect on cell growth. Cells (1 × 10^6^) were plated in 60-mm culture dishes and allowed to grow to ∼70% confluence. Next, they were treated with 500 pM of docetaxel and 50 nM of cytochalasin for 24 h, followed by washing with PBS and trypsinization to remove adherent cells. Exosome isolation was performed as per the manufacturer’s protocol (Total Exosome Isolation Kit, Invitrogen). Briefly, equal number of cells in culture media (drug treated and non-treated group) were centrifuged at 2,000*g* for 30 min, to remove cells and debris. The supernatant containing cell-free culture media was carefully transferred to new tubes without disturbing the pellet. To the new tubes, 0.5 volumes of Total Exosome Isolation reagent was added, mixed by vortexing and incubated at 4 °C for 12 h. After incubation for 12 h, the samples were centrifuged at 10,000*g* for 1 h at 4 °C. The supernatant was discarded and the pellets were resuspended in 1 × PBS. Total number of exosomes isolated from drug-treated and non-treated groups were analysed using NanoSight nanoparticle tracking analysis.

### Microarray study

DiL-Ac-LDL-labelled HUVEC and CFSE-labelled MDA-MB-231 cells were co-cultured in Matrigel using standard co-culture protocol for 36 h. The cells were harvested and stained with PECAM-1 (1:25) (Abcam) and Alexa Fluor 647 (1:100) (Life Technologies) secondary antibodies. Stained cells were then FACS sorted into PECAM-1+/DiL-Ac-LDL+/CFSE+ and PECAM-1+/DiL-Ac-LDL+/CFSE− populations, capturing HUVECs that did and did not receive nanobridge-mediated transfer. The sorted cells were then pelleted and snap frozen in liquid nitrogen. Total RNA isolation was carried out using the Ambion mirVana RNA Isolation Kit (Life Technologies) as per the manufacturer’s protocol and quantified using NanoDrop (Thermo Scientific). An miRNA microarray was carried out on two sets of the above mentioned samples from independent experiments with HUVECs stained with 7 μM CFSE as control. The Affymetrix GeneChip miRNA 3.0 Array was used. Labelling was carried out using the Affymetrix Flashtag Biotin HSR RNA labelling kit and standard protocol with a 1:500 dilution of ATP for Poly(A) Tailing. Hybridization was carried out using the Affymetrix GeneChip Hybridization Oven 640 for 42 h. Microarray data analysis was carried out using the bioinformatics toolbox in MATLAB (MathWorks); GEO accession number GSE72679.

### PCR reactions

p120RasGAP and glyceraldehyde 3-phosphate dehydrogenase (GAPDH) primers (IDT) were designed using messenger RNA reference sequences from NCBI database and Life Technologies OligoPerfect Designer software (GAPDH forward: 5′- AGTCAGCCGCATCTTCTTTT -3′, GAPDH reverse: 5′- GAGGTCAATGAAGGGGTCAT -3′; p120RasGAP forward: 5′- TAACAGCATTGGGGACATCA -3′, p120RasGAP reverse: 5′- TTGCCATCCACTGTGTCATT -3′).

Primer specificity was analysed using NCBI PrimerBLAST. Primer self-dimerization and hetero-dimerization were analysed using IDT OligoAnalyzer per SYBRGreen PCR assay experimental conditions. Co-cultured cells were FACS sorted. After total RNA extraction and quantification, complementary DNA was created using iScript cDNA Synthesis Kit (Bio-Rad) as per the manufacturer’s protocol. Real-time PCR was performed on MyiQ Real-time PCR Detection System (Bio-Rad) using iQ SYBRGreen Supermix (Bio-Rad) as per the manufacturer’s protocol for p120RasGAP and GAPDH primers.

*miRNA PCR assay*. Mono-cultured and co-cultured cells were FACS sorted. miRNAs were extracted from cells using the mirVana RNA isolation kit (Life Technologies), as per the manufacturer’s protocol, and quantified using the Take3 Micro-Volume plate (BioTek). cDNA was created using the Taqman miRNA Reverse Transcription kit (Life Technologies) as per the manufacturer’s protocol for RNU44 and hsa-mir-132 RT primers. PCR was performed using Taqman Universal PCR Master Mix II, no UNG (Life Technologies) and Taqman Small RNA Assay (Life Technologies) for RNU44 and hsa-mir-132 on MyiQ Real-Time PCR Detection System (BioRad).

### Statistical analyses

All statistical analyses were performed using Prism 6 (GraphPad). A Student’s *t*-test, one-way analysis of variance followed by Bonferroni’s test to compare different groups, or two-way analysis of variance was used to calculate statistical significance, with *P*-values<0.05 considered as significant.

## Additional information

**Accession codes:** The microarray data have been deposited in the GEO database under accession code GSE72679.

**How to cite this article:** Connor, Y. *et al*. Physical nanoscale conduit-mediated communication between tumour cells and the endothelium modulates endothelial phenotype. *Nat. Commun.* 6:8671 doi: 10.1038/ncomms9671 (2015).

## Supplementary Material

Supplementary Information Supplementary Figures 1-10, Supplementary Discussion and Supplementary References
