# Supplementary material for: Physical nanoscale conduit-mediated communication between tumour cells and the endothelium modulates endothelial phenotype
Source: Nat Commun. 2015 Dec 16;6:8671. doi: 10.1038/ncomms9671 (PMC4697439; doi:10.1038/ncomms9671)
Supplement: Supplementary Information — Supplementary Figures 1-10, Supplementary Discussion and Supplementary References [file ncomms9671-s1.pdf]

Supplementary figures

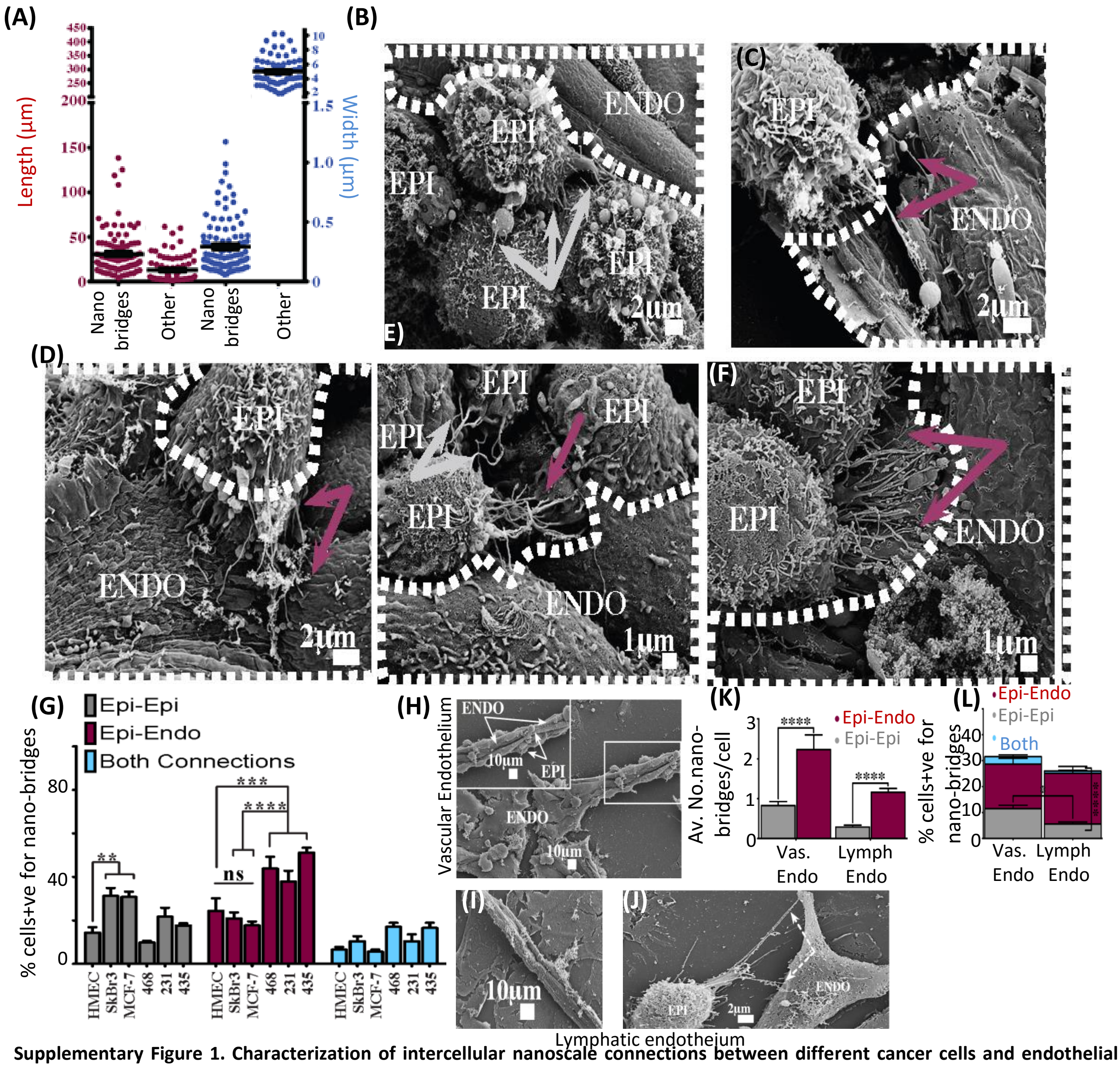

Supplementary Figure 1. Characterization of intercellular nanoscale connections between different cancer cells and endothelial cells from distinct sources. (A) Graph shows length and width of the nanoscale intercellular membrane bridges observed in this study vs larger cytoskeletal projections (other), such as lamellipodia and filopodia, as quantified using SEM. Representative SEM images show nanoscale heterotypic intercellular connections formed by (B) primary breast epithelial cells (HMEC), tumorigenic non-metastatic breast epithelial cell lines (C) SkBr3 and (D)MCF-7, and metastatic epithelial cell lines (E) MDA-MB-468 and (F) MDA-MB-435. Normal epithelial cells and tumorigenic non-metastatic cells equally, or indiscriminately, form monotypic (EPI-EPI) (maroon arrows) and heterotypic (EPI-ENDO) (gray arrows) nanoscale connections, while metastatic cells predominantly form epithelial-endothelial (EPI-ENDO) (maroon arrows) connections. (G) Graph shows percentage of cells that form homotypic or heterotypic connections or both. Quantification was performed on >300 cells of each cell type. (H) Metastatic breast cancer cells form nanoscale connections with primary human vascular and lymph endothelial vessels. SEM images capture interactions between MDA-MB-231 cells and primary human vascular and lymph endothelial vessels. Similar to phenotypes observed in 3D co-cultures with HUVEC vessels, MDA-MB-231 cells were observed elongating along the primary endothelial vessel structures (white arrows). There is significant vessel widening in vascular and lymphatic endothelial vessel co-cultures compared to monoculture. The lymph endothelial cells formed disorganized vessel structures with less cell-cell adhesion compared to vascular endothelial vessels, correlating with physiological difference between vascular and lymphatic endothelium. Nanoscale connections were formed between metastatic cells and primary human endothelium (dotted white arrows). (K-L) Graphs show MDA-MB-231 cells preferentially formed nanostructures with primary blood endothelial cells and primary lymph endothelial cells. Data shown are mean ± SEM. ****P<0.0001 (n>300).

(A)

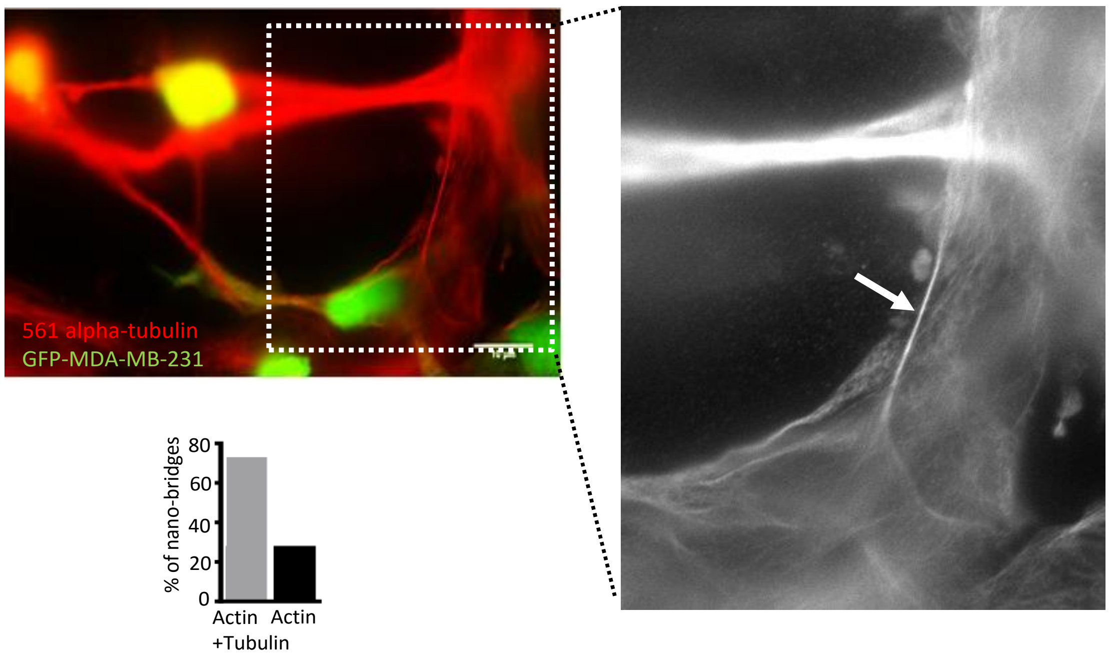

Supplementary Figure 2. Characterization of the heterotypic nanoscale membrane bridges and transfer between tumor and endothelial cells. (A) An epifluorescence image of a co-culture of GFP-expressing MDA-MB-231 cancer cell with endothelial tubules on Matrigel. The co-culture was stained with and antibody against tubulin. The inset shows a continuous tubulin-based composition. A population of the nanoscale membrane bridges were analyzed for composition. Graph shows the % of nanoscale membrane bridges that are composed of actin or actin+tubulin (n =.29).

(B)

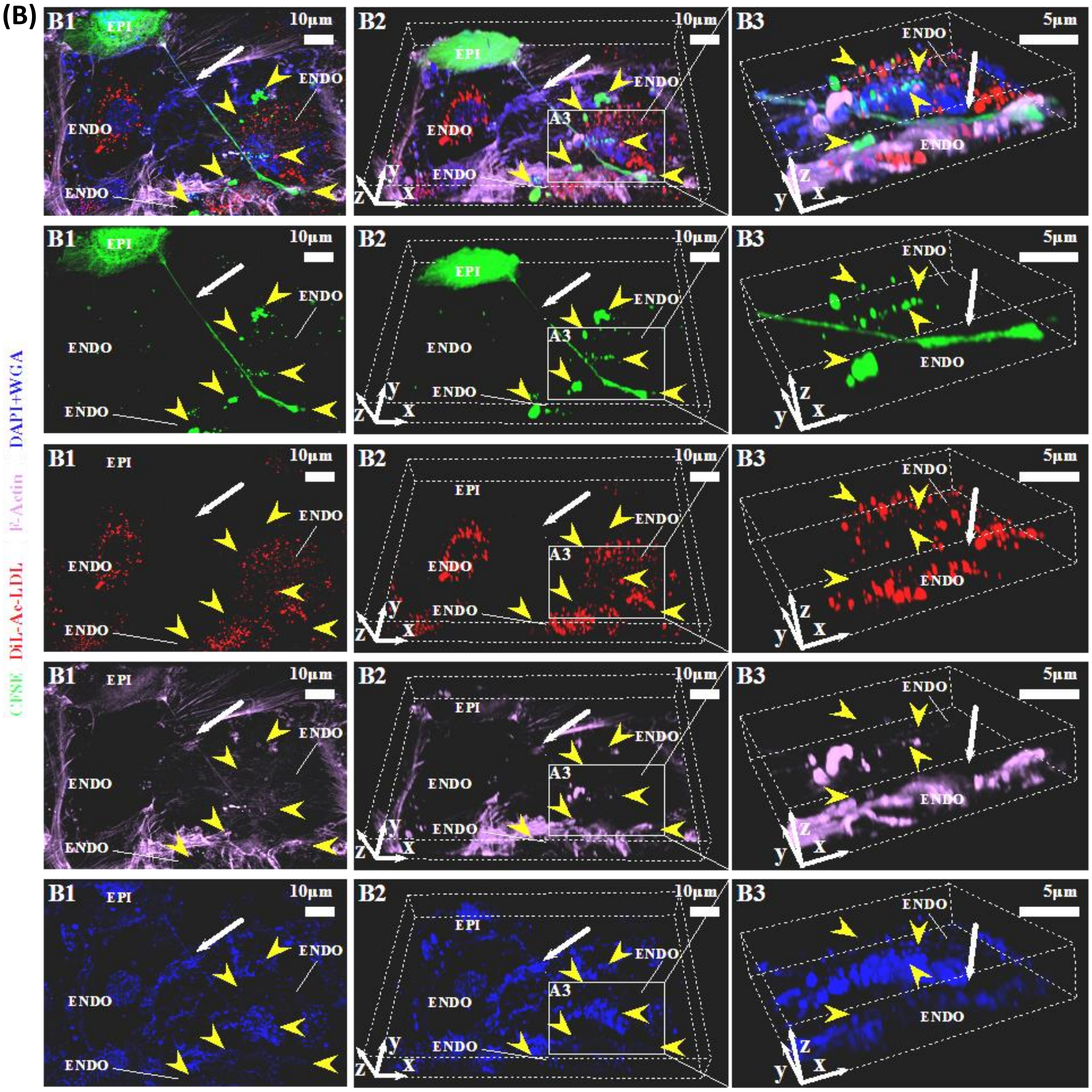

Supplementary Figure 2. Characterization of the heterotypic nanoscale membrane bridges and transfer between tumor and endothelial cells. (B) Confocal microscopy images capture nanoscale membrane bridges-mediated transfer of cytoplasmic contents. CFSE (green)-loaded MDA-MB-231 cells were co-cultured with the Dil-Ac-LDL(red)-labeled HUVECs. Transfer of the CFSE dye was observed after 24-hr co-culture (solid arrows). CFSE dye can be seen within HUVEC cells (yellow arrowheads).

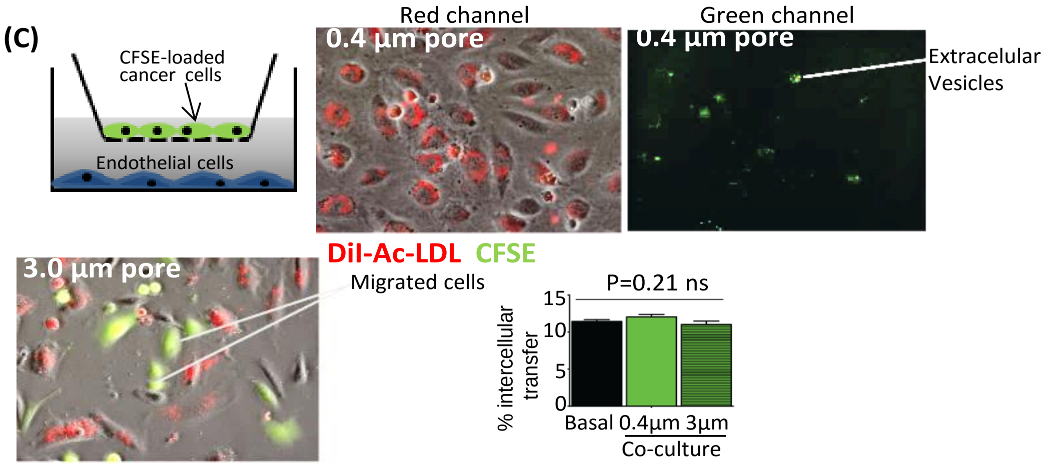

Supplementary Figure 2. Characterization of the heterotypic nanoscale membrane bridges and transfer between tumor and endothelial cells. (C) Schema shows the experimental set up. MDA-MB-231 cancer cells loaded with CFSE were cultured in upper insert of the Boyden chamber, and endothelial cells were cultured in the lower chamber. Epifluorescence images of endothelial cells labeled with DiI-Ac-LDL. While inserts with 3 µm pores allowed cancer cells to migrate to the lower chamber, no migration was evident in inserts with 0.4 µm pores, which however allowed extracellular vesicles to pass through. Graph shows the % of intercellular transfer of CFSE as quantified in the endothelial cells. Data shown are mean ± SEM (ANOVA followed by Bonferroni's post hoc test).

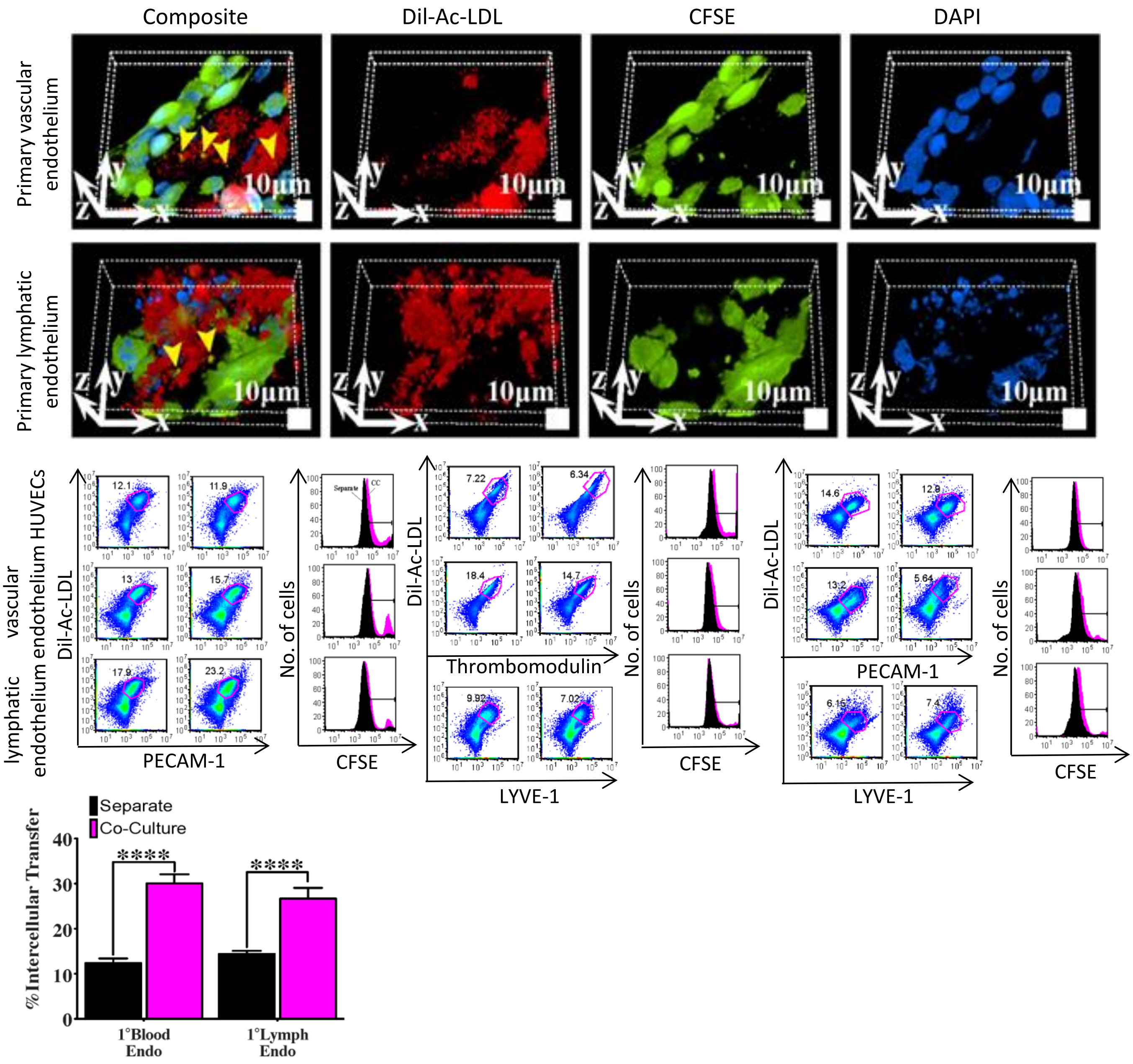

Supplementary Figure. 3. Nanoscale conduit-mediated intercellular transfer occurs between metastatic cells and primary vascular and lymphatic endothelial cells in the co-cultures (yellow arrow heads). Intracellular transfer was quantified using flow cytometry to determine the percentage of CFSE+ve cells in dual-labeled endothelial cell populations. Vascular endothelial cells were identified with immunostaining for PECAM-1/DiL-Ac-LDL, Thrombomodulin/DiL-Ac-LDL, or PECAM-1/Thrombomodulin. Lymph endothelial cells were identified with immunostaining for PECAM-1/DiL-Ac-LDL, LYVE-1/DiL-Ac-LDL, or PECAM-1/LYVE-1. Graph shows percentage of CFSE+ve endothelial cells. Data shown are mean ± SEM (n=3). ****P<0.0001 (ANOVA followed by Bonferroni's post hoc test).

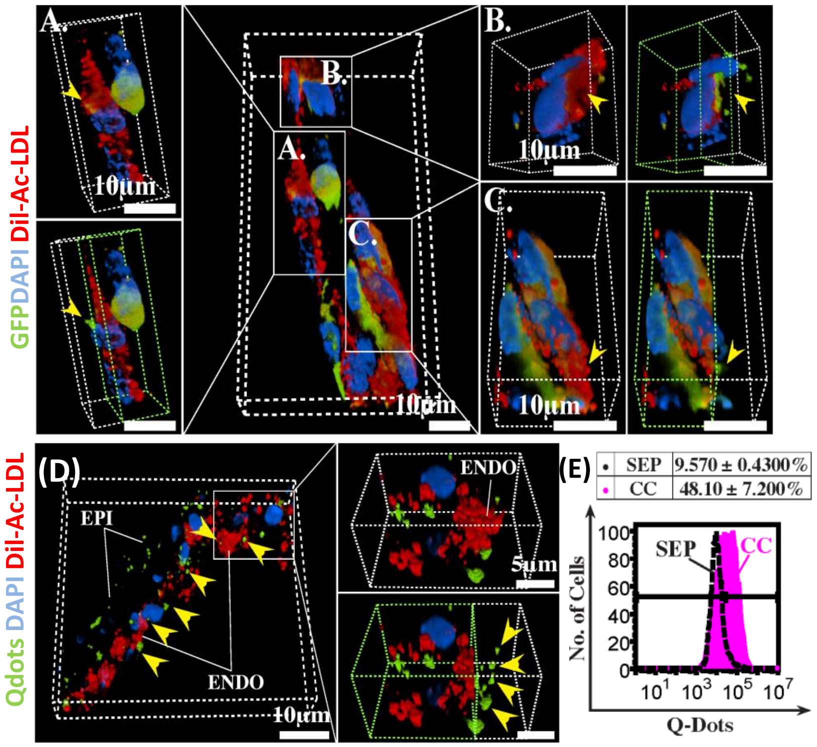

Supplementary Fig.4. Different types of payloads transferred by heterotypic intercellular connection. (A-B) Representative images show heterotypic intercellular transfer of proteins. Co-cultures with GFP-expressing MDA-MB-231 cells were established with HUVECs. Transfer of GFP was captured using fluorescent microscopy. 3D volume reconstruction illustrated transferred GFP in DiL-Ac-LDL (red) labelled endothelial cells (yellow arrowheads). Samples were counterstained with DAPI (blue). (D) 3D volume reconstruction illustrated transferred quantum dot nanoparticles in DiL-Ac-LDL (red) labeled endothelial cells. MDA-MB-231 cells were loaded with quantum dots and then used to establish the co-culture with endothelial cells. Yellow arrowheads indicate endothelial cells containing transferred nanoparticles (green). Quantification of intercellular transfer of Q-dots was confirmed via flow cytometry. In co-cultures, 48.10±7.2% of the endothelial cell population was positive for quantum dot compared to 9.570±0.43% in separate cultures in Boyden assay ($p<0.05$). Data shown is mean ± SEM.

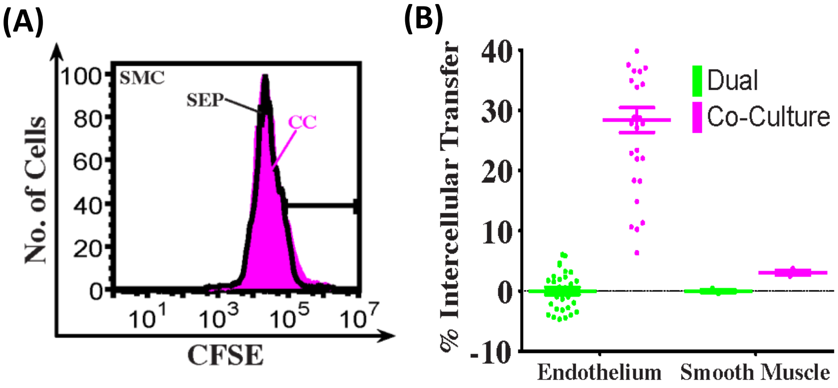

Supplementary Figure. 5. Metastatic cancer cells transfer CFSE to endothelial cells but not to vascular smooth muscle cells (VSMCs). CFSE-loaded MDA-MB-231 cells were incubated with HUVECs or VSMCs either in the co-culture or separately in Boyden chambers. The HUVECs and VSMCs were then sorted for the levels of CFSE transfer. Graph shows the quantification of transfer (Bar shows mean ± SE).

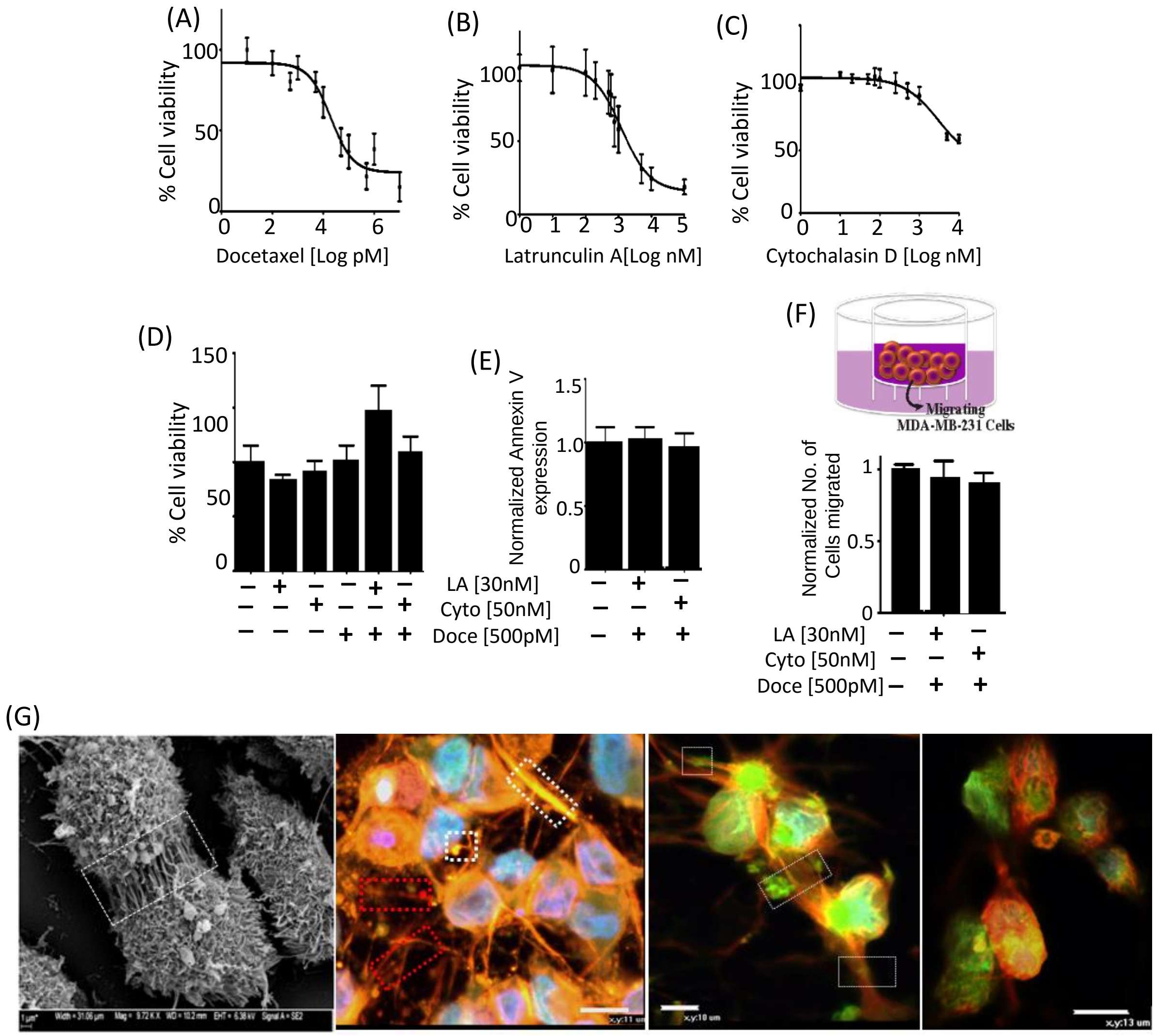

Supplementary Figure 6: Titration studies to identify concentrations of pharmacological modulators F-Actin and β-tubulin polymerization inhibitors that do not induce non-specific effects but inhibit the formation of heterotypic intercellular nanoscale conduits. (A-C) Concentration-effect curves for Docetaxel, Latrunculin A, and Cytoclasin D in MDA-MB-231 at 48 h. Viability was measured using MTS assay. (D) Concentrations that do not affect viability were used in combination studies, which revealed that at concentrations used the combinations of F-actin and β-tubulin polymerization disruptors showed no effect on cell proliferation at 48hrs or on (E) cell viability as assessed using Annexin V labeling. (F) Drug treatment did not affect cellular migration or invasion as assessed using Boyden chamber migration and invasion studies. Data shown are mean ± SEM (n=2-3 independent experiments). (G) A SEM images shows homotypic nanoscale connections between two MDA-MB-231 epithelial cells. The MDA-MB-231 culture was immunelabeled with an antibody against Cep55 (green), to delineate the cytokinesis bridges and counterstained with phalloidin-red to label actin. From left to right, the first confocal image shows a vehicle-treated culture with intact membrane bridges [red box] as well as cytokinesis bridges [white box]; the second image shows a culture treated with low concentration docetaxel + cytochalasin D, which disrupts the membrane bridges but cytokinesis bridges are intact; and the third micrograph shows a culture treated with high concentrations of the pharmacological inhibitors that disrupt both types of bridges.

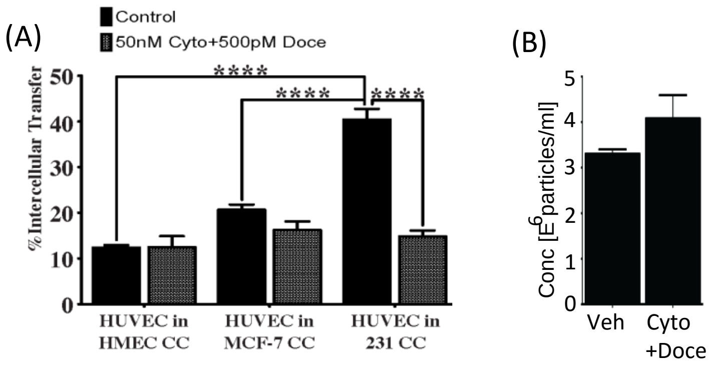

Supplementary Fig. 7. Effect of pharmacological inhibitors that disrupt nanoscale connections on heterotypic intercellular CFSE transfer. Co-cultures were established between HMECs, non-metastatic MCF7s and metastatic MDA-MB-231 cells with HUVECs. Baseline transfer between HMECs, non-metastatic MCF7 and endothelium was observed as compared with increased intercellular transfer between metastatic MDA-MB-231 and endothelium. (A) Graph shows pharmacological inhibition of heterotypic nanoscale connections inhibits this increase to baseline, but has no effect on baseline transfer. (B) Graph shows the quantification of exosome shedding by MDA-MB-231 cells treated with low concentration of docetaxel and cytochalasin. Data shown are mean ± SEM of 4 replicates from two independent experiments.

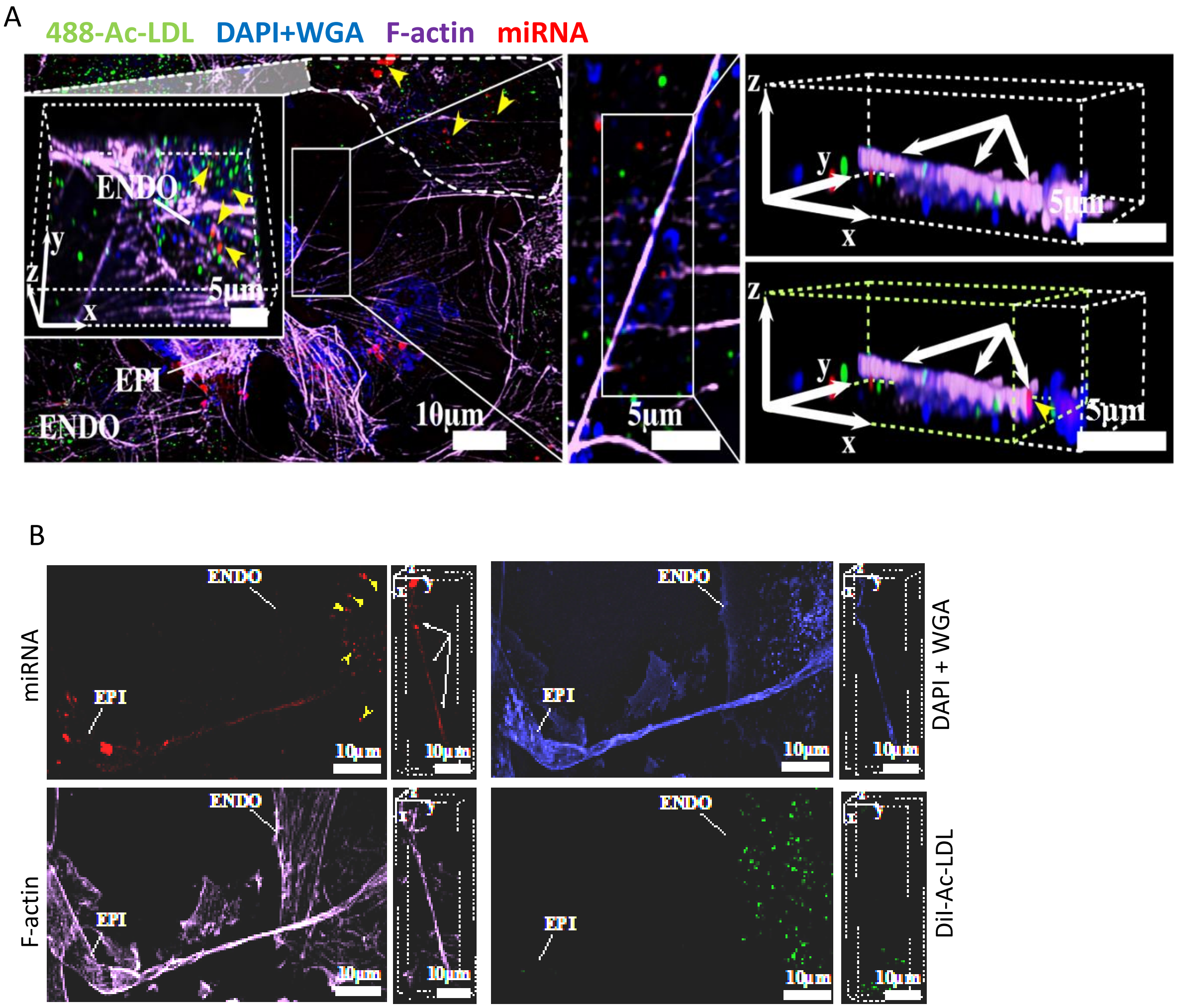

Supplementary Figure 8. The nanoscale connections act as conduits for intercellular transfer of microRNA between cancer and endothelial cells. (A) Representative confocal images show the transfer of Cy3-labeled control miRNAs from MDA-MB-231 cells (EPI) to endothelial cells (ENDO) at 36h of co-culture. Alexa fluor® 488-Ac-LDL (green) labeled endothelial cells were co-cultured with Cy3-labeled miRNA transfected MDA-MB-231. Co-cultures were counterstained with phalloidin (purple) and DAPI + WGA (blue). 3D-visualization shows the localization of miRNA within the nanoscale connections (white arrows), which act as conduits for horizontal transfer of miRNAs to endothelial cells (yellow arrow heads). (B) Images show separate channels for the merge image shown in Fig. 5A.

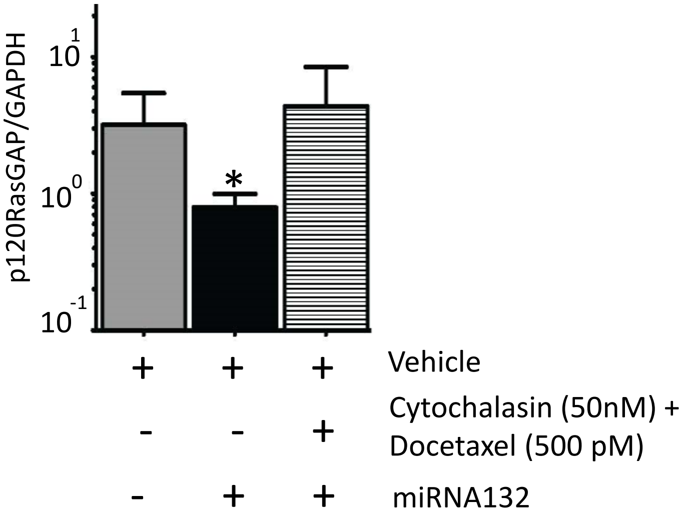

Supplementary Figure.9. Effect of pharmacological inhibition of nanoscale connections on downstream targets of miR132. Graphs show p120RasGAP expression in endothelial cells is decreased when cultured with miRNA-transfected MDA-MB-231 cells. Treatment of MDA-MB-231 cells with low concentration cytochalasin and docetaxel to inhibit the formation of nanoscale connections prevents the downregulation of p120RasGAP. Data shown are mean ± SEM. (*P<0.05. ANOVA followed by Bonferroni's post Hoc test).

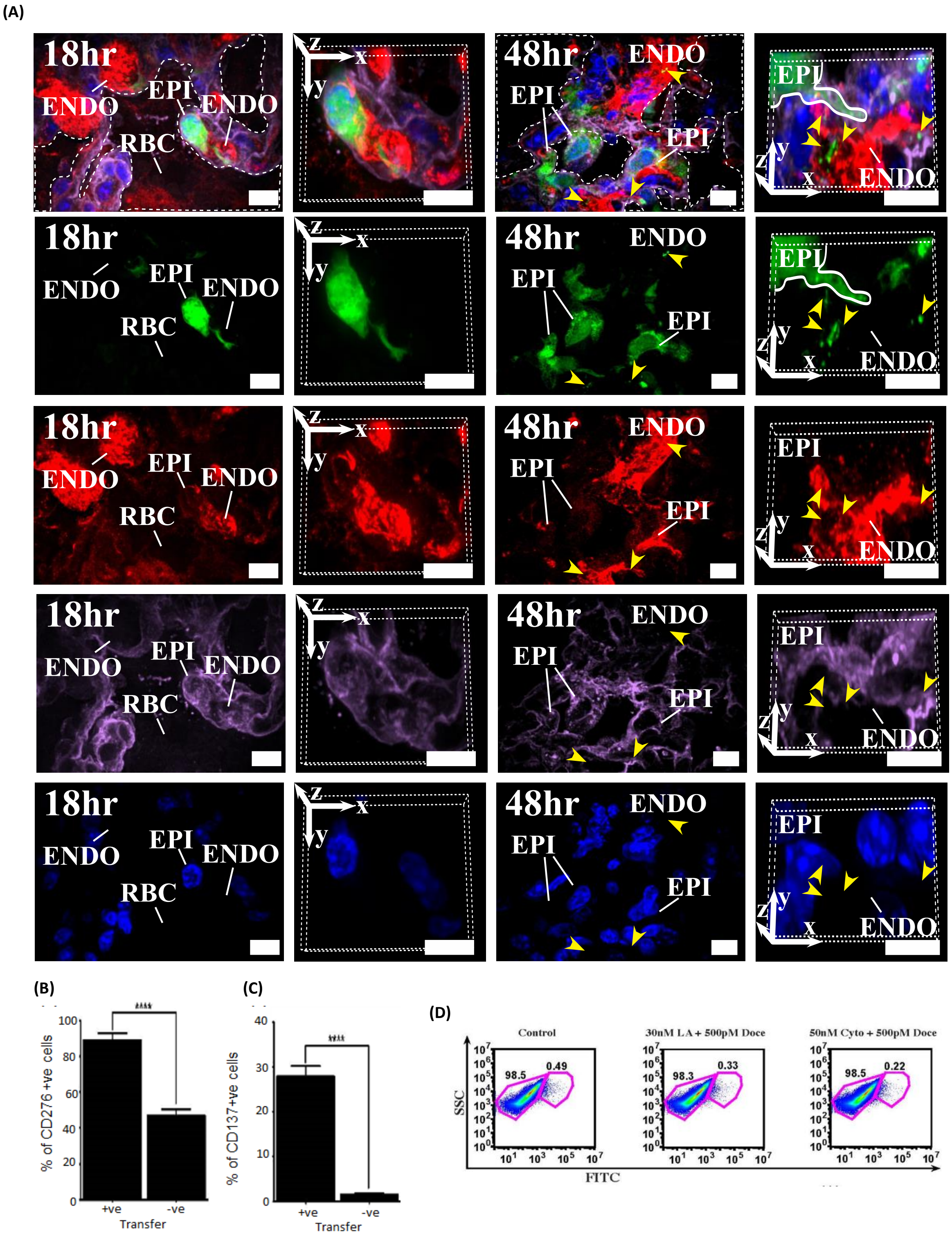

Supplementary Figure 10. Nanoscale conduit-mediated communication leads to increase in pathological angiogenic markers *in vivo.* (A) Confocal images show separate channels of the image in Fig. 7A. (B-C) Graphs show the expression of pathological angiogenic markers CD137 and CD276 in transfer +ve endothelial cells vs transfer –ve. Animals were injected with CFSE-loaded MDA-MB-231 cancer cells via the tail-vein and the lungs were excised at defined time points. Endothelial cells were isolated from the mouse lungs 48hrs post-injection. The endothelial cells were sorted into intercellular CFSE transfer+/-ve cell populations. The two populations were then stained for expression of CD276 and CD137. Graphs show significantly higher expression of pathological angiogenic markers in intercellular CFSE transfer+ve endothelial cells ($p<0.0001$). (D) Dot plots for transfer of CFSE from untreated and treated cancer cells to endothelial cells.

Supplementary discussion

Assumptions for building the mathematical model for the structure. We utilized a filopodial model[1] to calculate the buckling limits of thin, actin-only nanoscale intercellular projections such as TNTs. The maximum projection length for a given minimum diameter at the buckling limit[2] is plotted for actin-only TNTs (purple line). This curve is overlaid with the experimental length and diameter measurements from the observed thin projections measured in these studies. As shown in Fig. 2, projections containing only actin or projections containing both actin and tubulin can exist to the right of the curve (purple line). However, actin-only projections cannot exist to the left of the curve (green region). Actin-only projections will undergo structural failure (i.e. break) before they can achieve length scales in this region. However, there are several experimental data points which fall into the green shaded region (where actin-only structures cannot exist), suggesting that these structures must contain additional cytoskeletal proteins to achieve these length scales. The geometry of actin filaments is advantageous for tensile strength, but these filaments have lower flexural strength. In contrast, microtubules have a geometry that supports greater compressive and flexural loads. The mean flexural rigidity of microtubules is nearly 300 times that of actin filaments[3]. Extrapolating this model, we examined the effect of incorporating tubulin in these projections. The maximum length was plotted against the minimum diameter for varying fractions of tubulin incorporated in the projection. Addition of tubulin to the projections increases the overall flexural rigidity, shifting the curves left of the actin-only limit (purple line), thus allowing for longer and thinner nanoscale connections/conduits seen in the current study. However, due to the larger radius of microtubules (4x radius of actin filaments), there is an optimal fraction of tubulin that can be incorporated into the projection before the effect is reversed. There is an optimal tubulin fraction that will provide the desired rigidity without compromising the thickness. The optimal fraction of microtubules is about 6.6% (red dashed line) to maximize nanoscale tether flexural strength, while minimizing thickness. Tubulin fractions greater than this amount shift the curve to the right, reducing the maximum length to minimum diameter ratio and approaching the actin-only curve.

Known metastatic functions of miRNAs that are differentially regulated in recipient endothelial cells:
MicroRNA210, which was upregulated in recipient endothelial cells, has been shown to activate endothelial cells via down-regulation of ephrin A3, and facilitate metastasis[4]. Similarly, miR182 can down-regulate *Missing in Metastasis* protein and promote breast cancer metastasis via the activation of Rho[5]. MiR-34c can regulate the permeability of blood–tumor barrier via expression changes of ZO-1, occludin, and claudin-5[6]. MiR92a is also implicated in metastasis[7], and can promote endothelial cell migration in the presence of cancer cells[8]. Interestingly, we also observed an up-regulation of miR29b, which was shown to be a metastatic repressor[9], although it can activate endothelial cells[10,11]. In contrast, miR221 is implicated in driving an aggressive basal-like phenotype in breast cancer, although physiologically it can exert an angiostatic phenotype[12]. Indeed, it is increasingly becoming evident that a miR 'regulon' governs the phenotypic outcome rather than specific

miRNAs, and that the outcome is context- and cell-specific. We observed a distinct set of miRNAs that were downregulated in the recipient endothelial cells, consistent with previous studies where miRNAs have been reported to regulate miRNA expression[13].

Supplementary references

1**.** A. Mogilner, B. Rubinstein. The physics of filopodial protrusion. *Biophys. J*. **82**, 782-95 (2005)

2. J. Y. Shao, R. M. Hochmuth. Micropipette suction for measuring piconewton forces of adhesion and tether formation from neutrophil membranes. *Biophys. J*, **71**, 2892-902 (1996)

3. F. Gittes, B. Mickey, J. Nettleton, J. Howard. Flexural rigidity of microtubules and actin filaments measured from thermal fluctuations in shape. *J Cell Biol*. **120**, 923-34 (1993).

4. R. Heusschen, M. van Gink, A.W. Griffioen, V.L. Thijssen. MicroRNAs in the tumor endothelium: novel controls on the angioregulatory switchboard. *Biochim Biophys Acta.* **1805**, 87-96 (2010).

5. R. Lei, J. Tang, X. Zhuang, R. Deng, G. Li, J. Yu, Y. Liang, J. Xiao, H-Y. Wang, Q. Yang, G. Hu. Suppression of MIM by microRNA-182 activates RhoA and promotes breast cancer metastasis. *Oncogene* **33**, 1287-1296 (2014).

6. L. Zhao, P. Wang, Y. Liu, J. Ma, Y. Xue, miR-34c Regulates the Permeability of Blood–Tumor Barrier via MAZ-Mediated Expression Changes of ZO-1, Occludin, and Claudin-5. *J. Cell. Physiol*., **230**: 716–731 (2015)

7. M. Li, X. Guan, Y. Sun, J. Mi, X. Shu, F. Liu, C. Li, miR-92a family and their target genes in tumorigenesis and metastasis. *Exp. Cell Res*. **323**, 1-6 (2014).

8. T. Umezu, K. Ohyashiki, M. Kuroda, J.H. Ohyashiki. Leukemia cell to endothelial cell communication via exosomal miRNAs. *Oncogene.* **32**, 2747-55 (2013).

9. J. Chou, J.H. Lin, A.Brenot, J. Kim, S. Provot, Z. Werb, GATA3 suppresses metastasis and modulates the tumour microenvironment by regulating *microRNA-29b* expression. *Nat Cell Biol*. **15**, 201–213 (2013).

10. L-X. Yan, X-F, Huang, Q. Shao, et al. MicroRNA miR-21 overexpression in human breast cancer is associated with advanced clinical stage, lymph node metastasis and patient poor prognosis. *RNA* **14**, 2348-2360 (2008).

11.H-Q. Zhu, Q. li, L-Y. Dong, Q. Zhou, H. Wang, Y. Wang, MicroRNA-29b promotes high-fat diet-stimulated endothelial permeability and apoptosis in apoE knock-out mice by down-regulating MT1 expression. *Int J Cardiol* **176**,764-70 (2014).

12. M.Y. Shah, G.A. Calin. MicroRNAs miR-221 and miR-222: a new level of regulation in aggressive breast cancer. Genome Med. **3**: 56 (2011).

13. A. Tuccoli, L. Poliseno, G. Rainaldi, miRNAs regulate miRNAs: coordinated transcriptional and post-transcriptional regulation. *Cell Cycle*. **5**, 2473-6 (2006).
